# Multi-metal cooperation drives chemoresistance in lung cancer and is reversed by the membrane-permeable chelator MiADMSA

**DOI:** 10.1038/s41420-026-03222-8

**Published:** 2026-07-06

**Authors:** Hannah L. Richards, Steven J. Bell, Katherine E. V. Deck, Anaïs M. T. Y. Wiech, Emma Tarrant, Carissa M. Lloyd, Juan A. Aguilar, Dan Gelvan, William D. G. Brittain, Patricia A. J. Muller

**Affiliations:** 1 Department of Biosciences, Durham University, Durham, UK; 2 Department of Chemistry, Durham University, Durham, UK; 3 Pleco Therapeutics BV, Apeldoorn, The Netherlands

**Keywords:** Lung cancer, Cancer therapy

## Abstract

Elevated levels of transition metals are a common feature of solid tumours and are associated with poor clinical outcomes. However, tumour cells are exposed to complex metal mixtures rather than individual ions, and the functional consequences of such multi-metal exposure remain poorly defined. Here, we show that subtoxic combinations of metals cooperate to drive robust chemoresistance in lung cancer cells. This phenotype is not recapitulated by any single metal, demonstrating that resistance arises from coordinated multi-metal activity rather than individual metal effects. We further find that endogenous metal pools contribute to this response, and that metal-induced reactive oxygen species (ROS) are required but not sufficient, indicating that additional metal-dependent signalling mechanisms underpin chemoresistance. Because resistance emerges from collective metal activity, targeting individual metals fails to restore chemosensitivity. We therefore evaluated a pan-metal chelation strategy and identify monoisoamyl dimercaptosuccinic acid (MiADMSA) as a membrane-permeable chelator capable of targeting intracellular metal pools. Using complementary biochemical and cellular approaches, including a fluorinated derivative to track intracellular activity, we demonstrate that MiADMSA acts within cells to reverse metal-induced chemoresistance across multiple lung cancer models. Importantly, MiADMSA suppresses tumour growth in metal-exposed xenografts. Together, these findings identify multi-metal cooperation as a previously underappreciated driver of chemoresistance and establish intracellular pan-metal chelation as a potentially actionable strategy to restore chemotherapy sensitivity.

## Introduction

Humans are exposed to substantial levels of heavy metal ions through environmental pollution, contaminated groundwater, and smoking. Some metals, including copper, zinc, and magnesium, are essential for life. These endogenous metal ions function as enzyme cofactors [1] and are tightly regulated by specialised transporters and chaperones [2]. However, even essential metals can contribute to cancer development by promoting tumour growth or impairing immune responses [3]. In contrast, exogenous metals have no physiological role and may exert purely toxic effects.

In excess, both endogenous and exogenous metals stimulate the overproduction of reactive oxygen species (ROS), which damage DNA and lipid membranes [4]. While low ROS levels are necessary for normal cellular signalling and are tightly controlled in healthy cells, cancer cells exhibit disrupted redox homoeostasis. Tumour cells tolerate elevated ROS by upregulating antioxidant and detoxification systems, enabling survival under oxidative stress [5]. Increased concentrations of metal ions have been detected in tumour tissue, as well as in the serum and urine of cancer patients, and higher intracellular metal levels are frequently associated with poorer survival outcomes [6, 7].

Patterns of exposure influence both the type of cancer and the specific metals that accumulate. In lung cancer, elevated arsenic, chromium, nickel, lead, cadmium, and copper are commonly linked to cigarette smoke and air pollution [8]. Cigarettes contain particularly high levels of lead, cadmium, chromium, and copper [9]. Given the strong association between smoking and lung cancer, especially small cell lung cancer [10], it is unsurprising that elevated levels of these metals are detectable in cancer patients [11]. Moreover, heavy metal exposure may shape the mutational landscape of lung tumours, with smoke-associated mutation patterns correlating with elevated plasma metal levels [12].

Therapeutically, metal chelation has been explored in cancer, particularly by targeting individual metals such as iron and copper [13, 14]. Although copper chelation has shown promise in limiting breast cancer metastasis in some studies, most single-metal chelation strategies have not significantly improved survival or enhanced chemotherapy efficacy [15]. The cumulative impact of multiple metals has received far less attention.

We investigated a pan-metal chelator, MiADMSA, in the context of lung cancer. Unlike conventional chelators used for acute metal poisoning, which primarily remove metals from the bloodstream [16], MiADMSA is membrane-permeable and can act within tissues. Originally developed as a derivative of DMSA for cadmium poisoning [17], MiADMSA has demonstrated efficacy in rodents, promoting urinary and faecal excretion of cadmium, lead, chromium, and excess copper without significant toxicity [18]. In humans, it has shown chelating activity against iron, manganese, cobalt, and cadmium in patients with hepatic metal accumulation [19].

Our findings indicate that combined metal burden contributes to chemoresistance in lung cancer cells. MiADMSA reduced intracellular metal levels, reversed chemoresistance in vitro and in mouse models, and did not interfere with chemotherapy uptake. These results suggest that targeting multiple metals simultaneously may be more effective than single-metal approaches. MiADMSA therefore represents a promising candidate for combination therapy in lung cancer patients with heavy metal accumulation.

## Results

### Establishing a multi-metal cell model system

To investigate the impact of concurrent metal exposure on chemoresistance, we established a controlled multi-metal treatment model incorporating six metals (Mn, Cu, Zn, Cr, Cd, and Pb) that are commonly detected in tobacco smoke and air pollution and have individually been reported to accumulate in lung cancer patient samples of serum or lung tissue (Supplementary Table 1). While the precise composition and relative abundance of metals in human tumours remain incompletely defined, multiple studies indicate that co-exposure and co-accumulation of several transition and heavy metals are frequent, with the presence of three or more metals reported in the majority of non-small cell lung cancer cases [20]. Given this complexity, our aim was not to recapitulate a single patient-specific metallomic profile, but rather to model a reproducible multi-metal stress condition that captures the biologically relevant feature of simultaneous metal exposure.

**Fig. 1:**
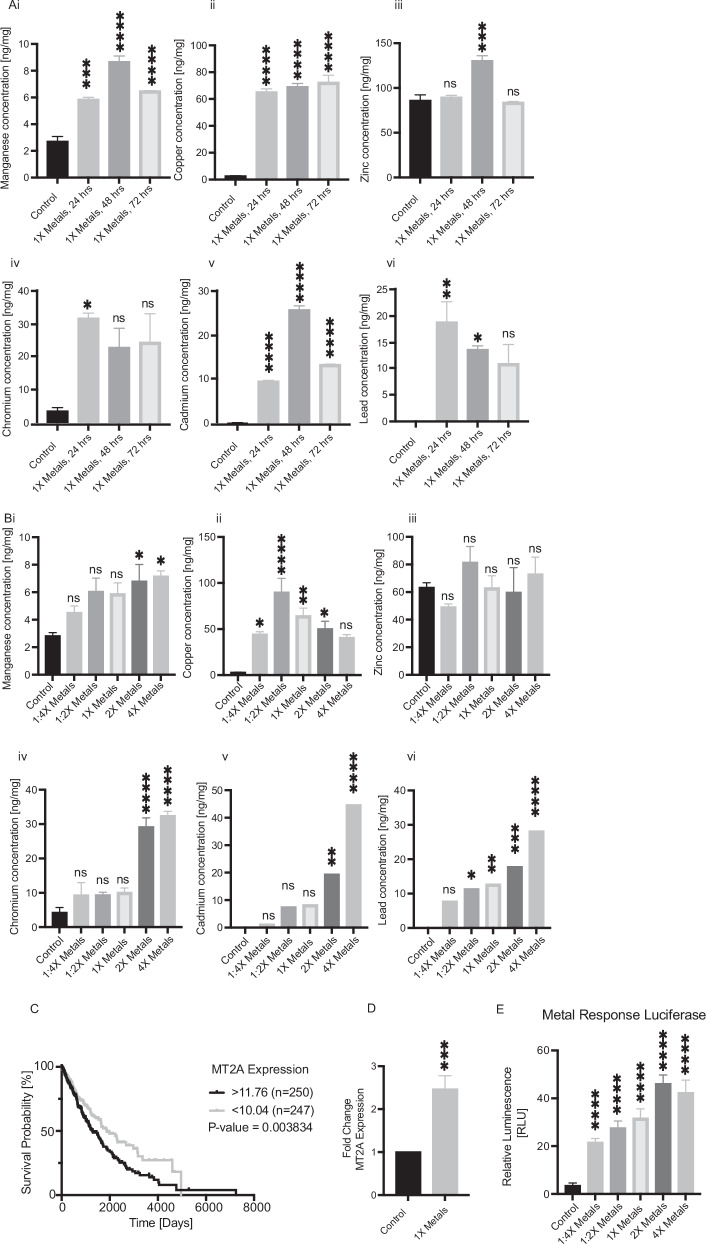
Metal accumulation in cancer cells. **A** ICP-MS to determine metal accumulation of individual metals in Beas-2B cells after being treated with mixed metal solution over time, *n* = 3. **B** ICP-MS to determine optimum dose of mixed metals for Beas-2B cells treated for 24 h, *n* = 3. **C** Kaplan–Meyer survival curve of TCGA lung cancer patients derived from Xenabrowser. Low and High gene expression of *MT2A* was analysed by using patient quartiles. **D** Gene expression of *MT2A* in Beas-2B cells treated with mixed metals for 24 h, determined by RT-qPCR, *n* = 3. **E** Beas-2B cells were transfected with a Metal Response luciferase (MRE) and TK renilla construct and incubated in mixed metal solution for 24 h. Luciferase values were measured and normalised for renilla expression *n* = 5. All error bars indicate SEM. *p < 0.05, **p < 0.01, ***p < 0.001, ****p < 0.0001.

Subtoxic levels for each metal ion were individually determined using viability assays in Beas-2B cells, a lung cell line that has previously been reported to gain oncogenic properties when exposed to metals [21]. IC_50_ and IC_10_ (a dose at which maximally 10% of cells are not surviving allowing us to determine a non-toxic starting point) values were determined for each metal (Fig. S1, Table S2). After trial and error in which we first combined metals at IC_10_ levels (Table S2) and then further reduced levels approximately 4-fold, we ended up with a mix that resulted in accumulation of all metal ions, without significantly decreasing viability when exposing cells for up to 72 h (Fig. 1A, Table 1 and Supplementary Fig. 2A). A 10X concentrated mix was freshly made for each experiment and used as 4X, 2X, 1:2X or 1:4X compared to the 1X mix.

**Table 1:** Metal salts and concentrations of the 6 metals mix used in majority of experiments.

Metal	Metal salt	Supplier	Parent solution concentration (mM)	Stock metal mix concentration (mM)	Concentration in 1X Metal Mix (µM)
Copper, Cu	CuSO_4_•5H_2_O	Honeywell	4.000	0.6400	5.0
Zinc, Zn	ZnSO_4_•7H_2_O	Merck	10.000	0.6400	5.0
Manganese, Mn	MnCl_2_•4H_2_O	Alfa Aesar	8.000	0.5120	4.0
Cadmium, Cd	CdCl_2_•2.5H_2_O	Alfa Aesar	0.650	0.0256	0.6
Chromium, Cr	K_2_Cr_2_O_7_	VWR	0.320	0.0640	0.5
Lead, Pb	(CH_3_COO)_2_Pb	Merck	0.65	0.256	2.0

Metals were dissolved in HNO_3_ as a 10X concentrated parent solution, diluted in water to a 1X solution and combined in concentrations as indicated in the stock metal mix, from which 1:4, 1:2, 1X, 2X and 4X concentrations were used as indicated in each figure.

Using ICP-MS, we noticed that for the 1X metal mix, five of the six metals were significantly accumulated intracellularly within 24 h when compared to control cells. Zinc levels only increased after 48 h. Interestingly, for most metal ions, levels were relatively stable over 24–72 h.

Next, to establish the optimal dose of metal ions, Beas-2B cells were exposed to concentrated as well as diluted versions of the metal mix for 24 h after which metal content was analysed (Fig. 1B). Manganese, chromium, cadmium and lead ions all showed dose-dependent increases in intracellular metal ion levels of each exposed metal, while copper ions peaked at dilutions of 1:2. Notably, zinc ion levels did not accumulate much, which likely reflects the very tight homoeostatic control of zinc levels in cells. In addition, low amounts of zinc, copper and manganese are present in the medium which are required for cells to grow.

Metal ions are known to bind to intracellular proteins and can be stored bound to buffering proteins such as metallothioneins (MTs). Metallothioneins maintain a cellular metal homoeostasis by sequestering and releasing a variety of metal ions [22]. Increased metallothionein gene levels can therefore indicate metal burden [23] and increased *MT2A* expression has been associated with decreased survival in non-small cell lung cancer patients and an increase in metastasis [24, 25], making expression of *MT2A* an attractive biomarker for metal load in cells. To validate previous studies in a different patient set, we used the TCGA lung cancer dataset in the Xenabrowser software and demonstrated as expected that patients with increased *MT2A* expression had a reduced survival of almost 3 years (Fig. 1C). Determining mRNA expression of *MT2A*, we could clearly see a 2.5-fold increase in *MT2A* expression upon metal exposure (Fig. 1D). We also used an metal Response element (MRE) luciferase reporter construct in which five metal response elements from MTF-1 are cloned upstream of luciferase. In response to binding zinc ions, MTF-1 translocates from the cytoplasm to the nucleus to drive expression of various genes, including metallothioneins [26]. In response to exposure to other metal ions, zinc ions that are bound to metallothioneins are released, which then bind MTF1 and induce transcription. It was therefore not surprising to see that predominantly zinc, cadmium and copper promoted luciferase expression (Supplementary Fig. 2B). Using this reporter, we could identify a dose-dependent increase in luciferase levels of 20-fold at 1:4X metal mix and about 40-fold in the 2X metal mix (Fig. 1E). Together these results demonstrate that exposure to a metal mix at sub-toxic levels causes intracellular metal accumulation and a metal signalling response.

Dose-determining ICP-MS experiments were also performed in A549 (non-small cell lung cancer, Supplementary Fig. 3A) and H1417 (Small cell lung cancer, Supplementary Fig. 3C, E) cells, showing increasing intracellular concentrations of metal ions upon metal ion exposure. However, looking at each metal level in H1417 cells compared to A549 or Beas-2B cells we noticed overall uptake of metals in H1417 and A549 cells was a bit lower than Beas-2B cells, and we therefore used doses of 2X Mix for A549 and 4X mix for H1417 cells in future experiments, which did not show impaired cell survival (Supplementary Fig. 3B, D).

### Multi-metal ion exposure causes resistance to doxorubicin

**Fig. 2:**
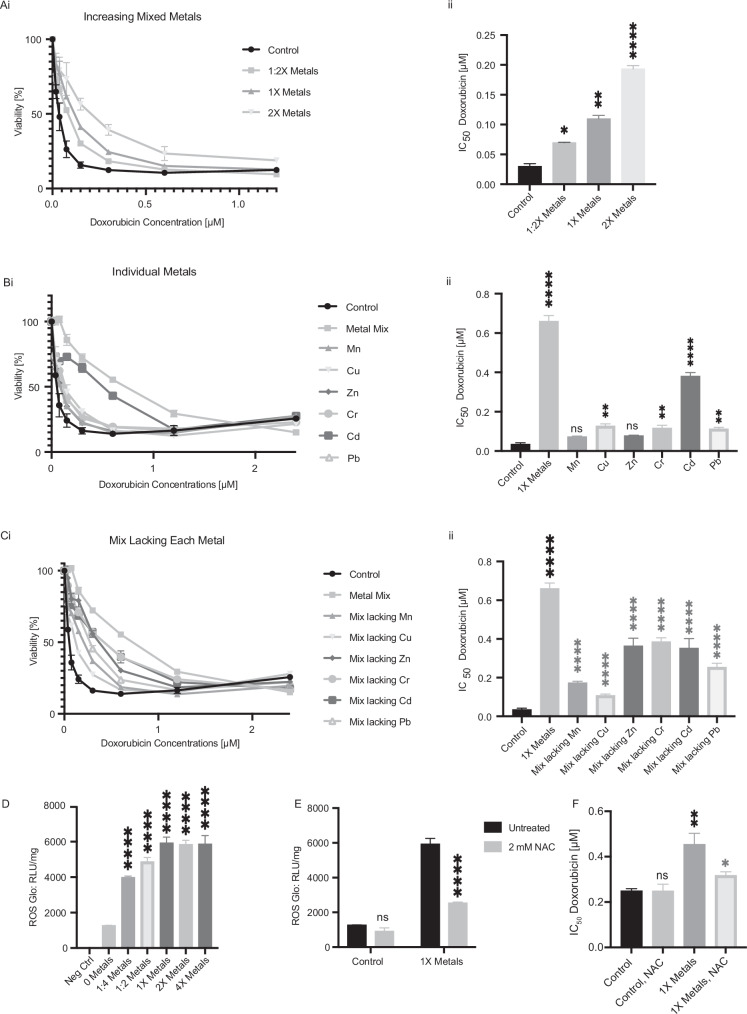
Multi-metal exposure causes doxorubicin resistance. **Ai** Resazurin survival curves of Beas-2B cells pre-incubated for 24 h with increasing concentrations of mixed metal solution followed by increasing doses of doxorubicin for 72 h. IC_50_ values were determined and plotted in **ii**, *n* = 3. **B** Resazurin survival curves of Beas-2B cells pre-incubated for 24 h with 1X dilutions of individual metals from the mixed metal solution followed by exposure to increasing doses of doxorubicin for 72 h. IC_50_ values were determined and plotted in **ii**, *n* = 3. **Ci** Resazurin survival curves of Beas-2B cells pre-incubated for 24 h with increasing concentrations of a mixed metal solutions, which were lacking one of the metals followed by exposure to increasing doses of doxorubicin for 72 h. IC_50_ values determined and plotted in **ii**, *n* = 3. **D** Measurement of ROS levels in A549 cells exposed to increasing concentrations of metals using a ROS luciferase readout (*n* = 3). **E** ROS levels in A549 cells upon metal exposure and NAC treatment (*n* = 3). **F** IC_50_ values derived from resazurin survival curves of A549 cells pre-incubated for 24 h with 1X mixed metal solution followed by exposure to 2 mM NAC and increasing doses of doxorubicin for 72 h. All error bars indicate SEM. *p < 0.05, **p < 0.01, ***p < 0.001, ****p < 0.0001 (black asterisks indicate statistics relative to control, grey relative to metal loaded conditions).

We next assessed if metal ion exposure changed the response of cells to chemotherapy. Beas-2B cells were cultured for 24 h in increasing metal mix solutions, after which cells were exposed to a concentration range of doxorubicin for 72 h (Fig. 2Ai). Results show that cells become less responsive to doxorubicin when co-exposed to increasing metal levels, quantified using IC_50_ values (Fig. 2Aii).

To determine whether all metals caused doxorubicin resistance or resistance was due to one individual metal ion, viability assays were performed with each metal ion in isolation at the dose used in the mix (Fig. 2Bi). No single metal ion increased resistance to the levels observed for the metal mix (Fig. 2Bii) or affected viability of cells significantly (Supplementary Fig. 4B). Of all metal ions, cadmium ions increased resistance most, by approximately 50%. Increases in intracellular levels of all metals were validated using ICP-MS (Supplementary Fig. 4D). Upon incubation with single metals, no significant changes in other metals were detected and the metal we had provided showed in most cases an increase in levels, except for zinc (Supplementary Fig. 4Diii). Notably, chromium and cadmium were not significantly raised in levels when we exposed cells to the metal mix using the multiple testing ANOVA on all samples. In a direct T-test, cadmium was significantly increased in levels compared to the control. There can be various reasons why differences exist in uptake of one metal in isolation or as part of a mix, but the most likely explanation is competition between metals.

To validate these results, we exposed Beas-2B cells to a metal ion mix in which one metal was lacking (Fig. 2Ci). Leaving out any one metal resulted in a significant decrease in chemoresistance (Fig. 2Cii) without affecting cell viability in the absence of doxorubicin significantly (Supplementary Fig. 4C). Interestingly, metal ion mixes lacking endogenous metal ions such as copper and manganese were most affected. Intracellular metal level analyses showed that removing one metal in the mix in general resulted in no accumulation of that metal, although some compensatory (co-) regulation or competition between metals may exist, which was particularly noted on cadmium ion levels when removing manganese, zinc or chromium (Supplementary Fig. 5). These results suggest that metal ions cooperate to lower sensitivity to chemotherapy.

### Metals may increase ROS to become insensitive to chemotherapy

Almost all metal ions are known to induce Reactive Oxygen Species (ROS) [27], prompting us to examine whether ROS contribute to metal-induced chemoresistance. Exposure of A549 cells to increasing concentrations of metal ions resulted in a 7–9-fold increase in intracellular ROS levels (Fig. 2D), accompanied by modest activation of ROS-responsive signalling, as indicated by *HO-1 (Heme Oxygenase 1)* expression (Supplementary Fig. 6A).

To determine if ROS participated in cell survival, we incubated cells in the ROS inhibitor NAC (N-Acetyl Cysteine), which works as ROS scavenger, reducer of disulphide bonds and precursor of glutathione biosynthesis [28]. Exposure to NAC reduced metal ion-induced ROS levels (Fig. 2E), and reduced metal-induced chemoresistance (Fig. 2F). As NAC has been shown to act as a weak chelator, we examined metal levels using ICP-MS and metal-response in MRE luciferase assays. NAC did not alter metal accumulation or metal-dependent reporter activity (Supplementary Fig. 6B, C), indicating that its effects are primarily due to ROS scavenging.

We next asked whether ROS magnitude, rather than the specific metal composition, determines chemoresistance. Expanding the metal mixture from 6 to 12 metals (while maintaining non-toxic concentrations; Table 2) did not further increase ROS levels (Supplementary Fig. 7A) or chemoresistance (Supplementary Fig. 7Bi), suggesting that a maximal ROS threshold had been reached. Exposure to a wider range of metals did not negatively impact initial viabilities (Supplementary Fig. 7Bii)

**Table 2:** Metal salts and concentrations of the 12 metals mix.

Metal	Metal Salt	Supplier	Parent solution concentration (mM)	Stock metal mix concentration (mM)	Concentration in 1X metal mix (µM)
Copper, Cu	CuSO_4_·5H_2_O	Honeywell	4.0	0.408	3.19
Zinc, Zn	ZnSO_4_·7H_2_O	Merck	10.0	0.408	3.19
Manganese, Mn	MnCl_2_·4H_2_O	Alfa Aesar	8.0	0.512	4.00
Cadmium, Cd	CdCl_2_·2.5H_2_O	Alfa Aesar	0.65	0.049	0.38
Chromium, Cr	K_2_Cr_2_O_7_	VWR	0.32	0.041	0.32
Lead, Pb	(CH_3_COO)_2_Pb	Merck	0.65	0.164	1.28
Vanadium, V	Na_3_VO_4_	Merck	0.8	0.033	0.26
Iron, Fe	C_6_H_8_O_7_⋅*x*Fe^3+^⋅*y*NH_3_	Alfa Aesar	8.0	0.326	2.55
Cobalt, Co	CoCl_2_·6H_2_O	Merck	6.5	0.288	2.25
Nickel, Ni	NiSO_4_·6H_2_O	Honeywell	6.0	0.816	6.38
Arsenic, As	NaAsO_2_	Merck	2.5	0.164	1.28
Mercury, Hg	HgCl_2_	SLS	0.3	0.244	1.91

Metals were dissolved in HNO3 as a 10X concentrated parent solution, diluted in water to a 1X solution and combined in concentrations as indicated in the stock metal mix.

Given that individual metals differ in their redox properties and roles in antioxidant defence, we assessed their relative contributions. While the metal mixture slightly reduced *SOD1* and *SOD2* expression, individual exposure to copper, manganese, or zinc increased *SOD1/2* expression (Supplementary Fig. 7C, D). Systematic omission of individual metals identified copper as the primary driver of ROS production (Supplementary Fig. 7E), consistent with higher ROS levels observed when cells are exposed to copper alone (Supplementary Fig. 7E). Removal of copper reduced doxorubicin resistance without affecting viability (Supplementary Fig. 7Fi–ii), confirming its contribution to chemoresistance. However, copper alone did not confer resistance (Supplementary Fig. 7Fi) or affect viability (Supplementary Fig. 7Fii), indicating that ROS generation is necessary, but not sufficient. Further supporting this, removal of manganese did not significantly affect ROS levels, but reduced chemoresistance (Supplementary Fig. 7E, Fi), demonstrating that ROS levels alone do not fully predict the resistant phenotype.

If additional metal-dependent signalling is important to drive chemoresistance, we reasoned that other ROS inducers may not be as potent as the metal mix to promote chemoresistance. We first used hydrogen peroxide and noted that a dose of 75 μM caused a similar amount of ROS as the 1:4X metal mix (Supplementary Fig. 8A). Using this dose in survival assays resulted in a moderate, but significant increase in doxorubicin resistance similar to the 1:4X metal mix (Supplementary Fig. 8Bii). Unlike metals, higher doses of hydrogen peroxide did induce more ROS, but resulted in a loss of cell viability (Supplementary Fig. 8Bi).

We next titrated in another ROS inducer menadione and noticed that low doses of menadione (3-10 μM) induced doxorubicin resistance (Supplementary Fig. 8Dii). These doses hardly caused any ROS and only 20 μM induced levels of ROS comparable to metals (Supplementary Fig. 8C). This concentration, however, was not viable long enough to perform survival assays (Supplementary fig. 8Di). These data suggest that H_2_O_2_ may induce ROS in a similar manner to metals and contribute to chemoresistance, albeit more potently resulting in cell death. In contrast, menadione is causing chemoresistance at a lower dose in a ROS-independent manner. Together, these findings indicate that while ROS contribute to metal-induced chemoresistance, they are not sufficient on their own. Instead, metals appear to promote resistance through a combination of ROS generation and additional metal-dependent signalling mechanisms.

### Several lung cancer cell lines become insensitive to a variety of chemotherapy in the presence of metals

**Fig. 3:**
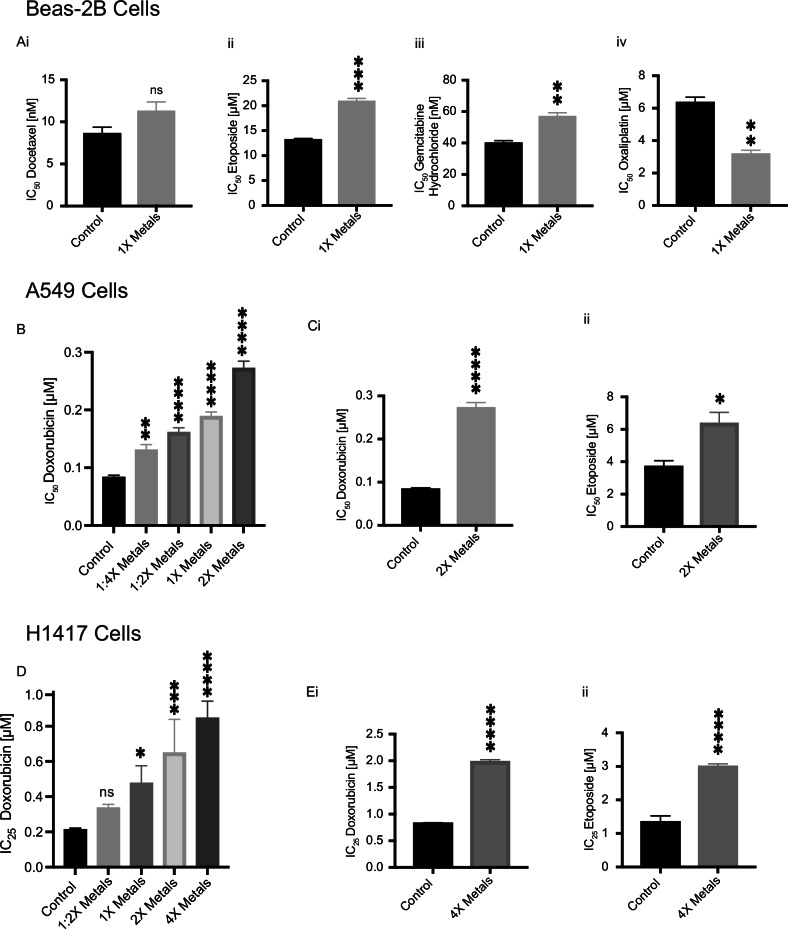
Metals induce chemoresistance in multiple cells to multiple drugs. **A** IC_50_ values of Beas-2B pre-exposed to a 1X metal mix for 24 h followed by exposure to docetaxel (**i**), etoposide (**ii**), gemcitabine hydrochloride (**iii**) and oxaliplatin (**iv**) for 72 h. **B** IC_50_ values of A549 cells pre-exposed to increasing doses of metal mix for 24 h followed by exposure to doxorubicin for 72 h. **C** IC_50_ values of A549 cells pre-exposed to a 2X metal mix for 24 h followed by exposure to **i** doxorubicin or **ii** etoposide for 72 h. **D** IC_25_ values of H1417 cells pre-exposed increasing concentrations of metal mix for 24 h followed by treatment with increasing doses of doxorubicin for 72 h. **E** IC_25_ values of H1417 cells pre-exposed to a 4X metal mix for 24 h followed by exposure to **i** doxorubicin or **ii** etoposide for 72 h. Error bars represent SEM. All experiments *n* = 3, *p < 0.05, **p < 0.01, ***p < 0.001, ****p < 0.0001.

Doxorubicin was one of the first chemotherapies used in lung cancers [29], but other drugs are now more frequently used to treat NSCLC and SCLC [30]. To determine if other chemotherapies are affected by metals, we assessed docetaxel, etoposide, oxaliplatin and gemcitabine in Beas-2B cells. Beas-2B cells became resistant to etoposide and gemcitabine, but not to oxaliplatin (Fig. 3Ai–iv). Resistance to docetaxel was marginal and not significant (Fig. 3Ai). Besides doxorubicin, etoposide showed the biggest and most significant difference in chemoresistance upon metal loading in Beas-2B cells (Fig. 3Aii). We next tested chemoresistance in A549 and in H1417 cells. In line with an increased metal loading (Supplementary Fig. 3A, C), a decreased sensitivity to doxorubicin and etoposide was seen for both lines when exposed to metal ions (Fig. 3B, D). Notably, IC_25_ values were used for H1417 in which a 25% reduction in viability was measured rather than 50%. H1417 are a suspension cell line resulting in a baseline that exceeded 50% survival (Supplementary Fig. 3E). Overall, the results here suggest that the metal ion mix confers chemoresistance to multiple chemotherapies with some selectivity, but across different lung cancer cell lines. Differences in metal sensitivity may reflect differences between cell lines and ways in which cells become resistant to chemotherapy in response to metal ion exposure.

### Metal ion-induced insensitivity to doxorubicin can be reversed using MiADMSA

**Fig. 4:**
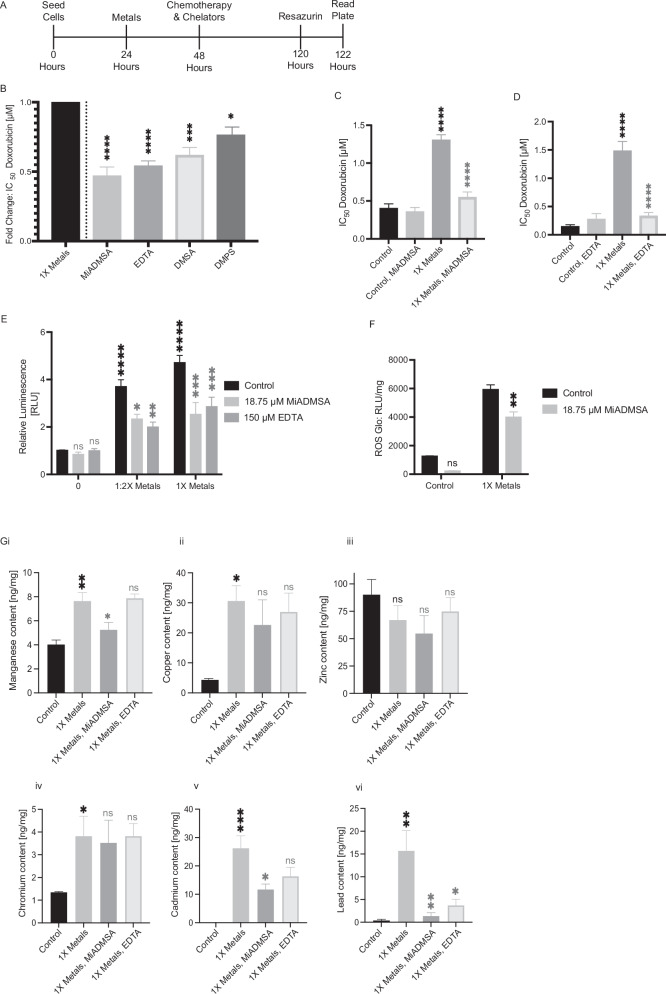
Chelation by MiADMSA reverses doxorubicin resistance. **A** Timeline of metal exposure and treatment with chemotherapy and chelators for resazurin assays. **B** Fold change IC_50_ values derived from resazurin survival curves of Beas-2B cells pre-incubated for 24 h with 1X metal mix followed by treatment with doxorubicin in conjunction with chelators (18.75 µM MiADMSA, 150 µM EDTA, 150 µM DMSA or 150 µM DMPS) for 72 h, *n* = 7. **C** IC_50_ values derived from resazurin survival curves of Beas-2B cells pre-incubated for 24 h with mixed metal solution followed by exposed to 18.75 µM MiADMSA in conjunction with increasing doses of doxorubicin for 72 h, *n* = 7. **D** IC_50_ values derived from resazurin survival curves of Beas-2B cells pre-incubated for 24 h with mixed metal solution followed by exposure to 150 µM EDTA in conjunction with increasing doses of doxorubicin for 72 h, *n* = 5. **E** Beas-2B cells were transfected with a Metal Response luciferase (MRE) and TK Renilla construct and incubated in mixed metal solution for 24 h followed by exposure to MiADMSA or EDTA for an additional 24 h. Luciferase values were measured and normalised for Renilla expression *n* = 3. **F** ROS levels were measured in Beas2B cells in response to metal exposure and MiADMSA treatment. **G** ICP-MS to determine the metal levels of Beas-2B cells treated for 24 h with 1X metal mix followed by exposure to 18.75 µM MiADMSA or 150 µM EDTA for an additional 24 h, *n* = 3. All error bars indicate SEM. *p < 0.05, **p < 0.01, ***p < 0.001, ****p < 0.0001 (black asterisks indicate statistics relative to control, grey relative to metal loaded conditions).

As the resistance we see is fast, 24 h after metal exposure, we believe that metal-induced resistance may be independent of mutations and may thus be reversible. To prove this, we used a variety of chelators that can bind several divalent metals. We tested EDTA, DMSA, DMPS and MiADMSA on Beas-2B cell viability. Cells were preincubated with the metal mix for 24 h, before doxorubicin was added with chelators (Fig. 4A). All chelators were used at concentrations at which we could detect a decrease in metal levels and at which no significant toxicity was observed. For EDTA, DMSA, DMPS this was at 150 μM, but for MiADMSA we could use a concentration as low as 18.75 μM. Of all chelators, MiADMSA was the most effective especially when considering this lower concentration (Fig. 4B). Effective dose was measured for each and an example of EDTA is shown in Supplementary Fig. 9A. Optimal effects of MiADMSA were seen at doses as low as 18.75 μM (Fig. 4C). Neither 150 μM EDTA or 18.75 μM MiADMSA impaired viability (Supplementary Fig. 9B, C) or affected chemotherapy treatment in the absence of metals (Fig. 4C, D, second bar). To assess the effect of chelation on intracellular metal levels, we used MRE luciferase assays or ICP-MS to look at individual metals. Both MiADMSA and EDTA were able to repress MRE luciferase response (Fig. 4E). Looking at metals individually, MiADMSA reduced manganese, cadmium and lead levels significantly, but EDTA only lowered lead ions significantly, despite being used at a much higher dose (Fig. 4G). We considered the possibility that metal ions prevented doxorubicin uptake, but if anything, metal ions appear to enhance uptake of doxorubicin (fluorescent itself) (Supplementary Fig. 9D, E) and chelation did not affect doxorubicin uptake. Finally, we assessed ROS levels and identified that MiADMSA lowered intracellular ROS levels in response to metals (Fig. 4F) and was able to reduce ROS dependent signalling as measured by *HO-1* expression (Supplementary Fig. 9F).

### MiADMSA has affinity for most of the metals in our mix, is lipid-soluble and works intracellularly

**Fig. 5:**
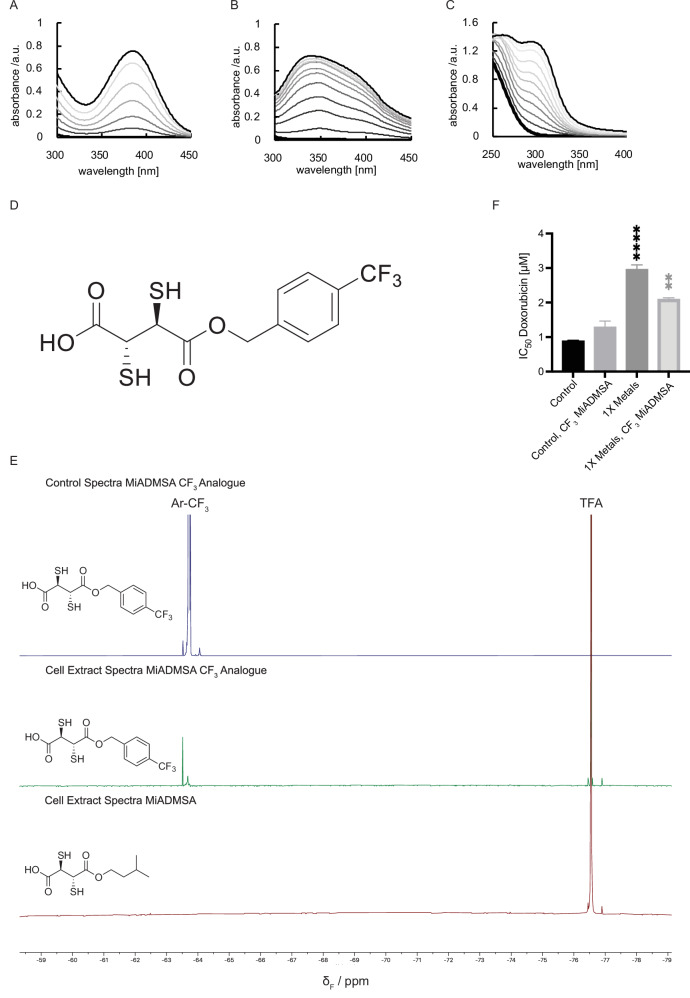
MiADMSA binds metals and works intracellularly. Evolution of UV-Vis spectral features upon titration of MiADMSA with **A** copper (0–0.3 equiv.), **B** manganese (0–0.6 equiv.), and **C** lead (0–0.6 equiv.). See supporting information for full spectra. **D** Structure of fluorinated (CF_3_) analogue of MiADMSA. **E** Fluorine NMR spectra of detected Fluorine MiADMSA analogue in Beas-2B cells. **F** IC_50_ values derived from resazurin survival curves of Beas-2B cells pre-incubated for 24 h with 1X mixed metal solution followed by exposure to 18.75 µM CF3 MiADMSA analogue in conjunction with increasing doses of doxorubicin for 72 h, *n* = 3. Error bars represent SEM. *p < 0.05, **p < 0.01, ***p < 0.001, ****p < 0.0001 (black asterisks indicate statistics relative to control, grey relative to metal loaded conditions).

Despite the growing biomedical prevalence of thiol chelating agents, literature reports characterising their metal binding properties are surprisingly scarce [31]. To the best of our knowledge, we herein report the first instance of metal-binding titrations for MiADMSA. UV-Vis spectroscopy was used to investigate the direct interactions between MiADMSA and the individual metal ions found in the metal mix. In the absence of any metals, MiADMSA exhibits a single strong absorption band centred around 250 nm, attributed to π → π* transitions of the carbonyl groups. Titration with copper, manganese, or lead ions at physiological pH led to the appearance of strong spectral features in the range of 300–400 nm (Fig. 5A–C), characteristic of metal-thiolate LMCT transitions arising from the formation of metal-ligand complexes. Binding constants were subsequently estimated by fitting the observed absorbance changes at selected wavelengths to a 2:1 (L:M) BindFit model (see supporting information). In the case of zinc and cadmium ions, the spectral changes observed upon titration of the metal into MiADMSA were notably weaker in comparison (Supplementary Figs. 10–12). This is most likely due to the d^10^ electronic configuration of both divalent metal ions, which precludes d-d transitions, and greatly limits ligand-metal transitions due to the lack of suitable acceptor orbitals. Therefore, while approximations of binding constants were still obtained for zinc and cadmium (Supplementary Table S3), the higher percentage errors should be noted. Unfortunately, observation of chromium ion binding was found to be incompatible with our method, although this does not necessarily exclude the possibility of its binding to MiADMSA.

Using Chemicalize (https://chemicalize.com/, accessed September 2025), a platform developed by ChemAxon, we calculated the physicochemical properties of MiADMSA, DMSA, EDTA and DMPS all in their neutral forms. LogP values were calculated as follows MiADMSA = 2.016, DMSA = 0.259, EDTA = −1.879 and DMPS = −0.078. LogD7.4 values were calculated as follows MiADMSA = −1.925, DMSA = −5.413, EDTA = −14.792 and DMPS = −2.518. These values point to the ester moiety within MiADMSA being critical in influencing the lipophilicity of the chelator. Out of all the interrogated compounds it was seen that MiADMSA is significantly more lipid soluble than other related chelators.

Our data suggests that MiADMSA works intracellularly after metal loading to extract metals from cells rather than limit metal ion uptake. To investigate if dimercaptosuccinic acid mono-esters can work intracellularly, we generated a fluorine-containing CF_3_ analogue of MiADMSA (Fig. 5D). Fluorine is a chemical element that does not naturally occur in cells but is spin active and has a wide chemical shift window, thus allowing for spectroscopic interrogation [32, 33]. We designed the analogue to incorporate a CF_3_ group which would give a strong singlet within the NMR making it most useful for working at low concentrations. In addition, due to the lack of commercially available aliphatic branched chain fluorinated alcohols we also decided to use a benzyl alcohol derivative. Using ^19^F NMR, we could clearly see a peak in the ^19^F NMR spectrum that correlates to the fluorine analogue, but not the original MiADMSA that did not contain fluorine. Most interestingly, the fluorine peak was also detected in the cell lysate (Fig. 5E), suggesting that the MiADMSA CF_3_ analogue was present intracellularly. Analogous compounds can have limitations in drawing direct conclusions about the parent compound, however in this case it was found that the MiADMSA CF_3_ analogue was still effective in restoring chemoresistance in Beas-2B cells (Fig. 5F). Lipophilicity calculations on the analogue were also conducted giving a LogD7.4 = −1.263 showing that we had selected a molecule with a similar partition coefficient to our parent compound (MiaDMSA LogD7.4 = −1.925).

### MiADMSA works to restore chemosensitivity in NSCLC and SCLC

**Fig. 6:**
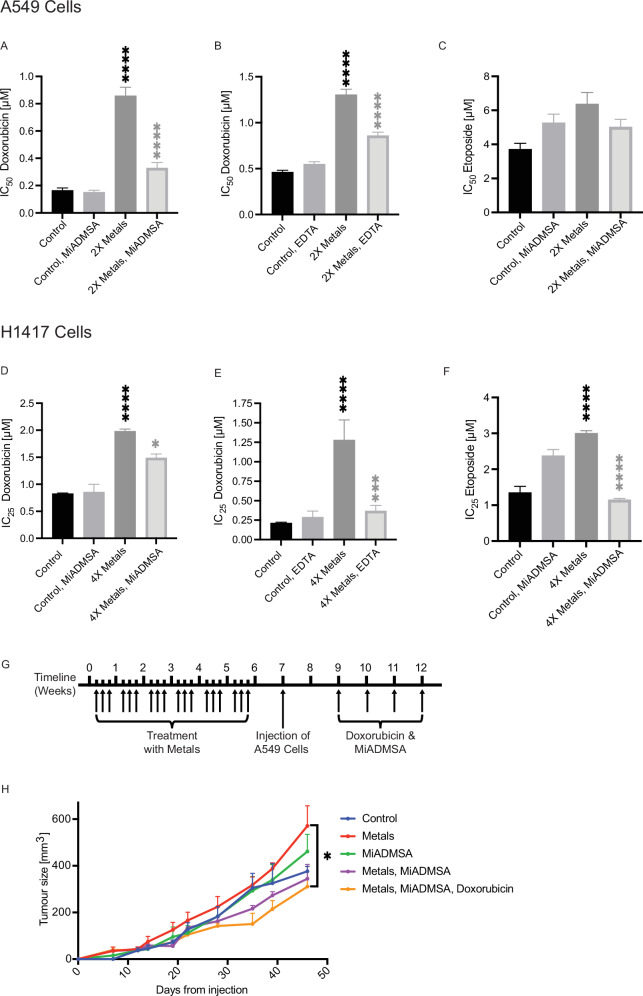
Chelation restores chemoresistance in cancer cells in vitro and in vivo. IC_50_ values derived from resazurin survival curves of A549 cells pre-incubated for 24 h with 2X mixed metal solution followed by exposure to **A** MiADMSA and doxorubicin, **B** EDTA and doxorubicin or **C** MiADMSA and etoposide. *n* = 3. IC_25_ values derived from resazurin survival curves of H1417 cells pre-incubated for 24 h with 4X mixed metal solution followed by exposure to **D** MiADMSA and doxorubicin, **E** EDTA and doxorubicin or **F** MiADMSA and etoposide. *n* = 3. **G** Timeline indicating metal treatment, xenograft injections, doxorubicin and MiADMSA treatment in mice. Error bars represent SEM. *p < 0.05, **p < 0.01, ***p < 0.001, ****p < 0.0001, black asterisks indicate statistics relative to control, grey relative to metal loaded conditions. **H** A549 xenografted tumours in mice were measured using calliper and plotted for tumour growth in time. The area under curve was calculated, and statistically significant changes were calculated using a one-way ANOVA in Prism 11. Error bars indicate SD.

We finally assessed the use of MiADMSA in lung cancer cell lines, and we could completely restore doxorubicin sensitivity in A549 cells (Fig. 6A) and significantly reduce resistance in H1417 cells using MiADMSA (Fig. 6D) without reducing cell viability (Supplementary Fig. 13). EDTA was also able to restore sensitivity, but at the higher dose of 150 μM and less effectively in A549 cells (Fig. 6B, E). MiADMSA was also very effective in restoring etoposide chemosensitivity in H1417 (Fig. 6F), but did not significantly reduced resistance in A549 cells (Fig. 6C), potentially reflecting the fact that etoposide is less effective in NSCLC compared to SCLC. To validate that MiADMSA can be active in vivo, we used xenograft experiments in which we pre-exposed mice to a metal mix for 6 weeks, followed by injecting A549 cells subcutaneously (Fig. 6G). Mice were subsequently exposed to doxorubicin and MiADMSA once per week until tumours started to show signs of necrosis. Interestingly, tumours appeared to grow fastest in the presence of metals and MiADMSA was most effective to reverse tumour growth when combined with doxorubicin (Fig. 6H).

In conclusion, we believe these data demonstrate that MiADMSA is a drug that can be used for restoring chemosensitivity to chemotherapeutics in those patients that are experiencing metal accumulation.

## Discussion

Multiple studies have reported that metal ions accumulate in tumour tissue [6, 34]. However, most experimental cancer models examine only one or two metals at a time, which may not reflect the complex metal burden present in patients. Our findings show that combined metal ion accumulation can reduce chemotherapy efficacy by up to fivefold. Notably, this reduction occurred within 24 h, indicating that resistance was unlikely due to new mutational events. Instead, the total metal burden appeared more important than any single metal alone. This observation has implications for how safe exposure limits are defined, as metals are typically assessed individually rather than in combination.

Among the chemotherapies tested, doxorubicin was most affected by metal exposure, followed by etoposide and gemcitabine. Doxorubicin is an anthracycline, while etoposide and gemcitabine are classified as antimetabolites [35]. These agents disrupt DNA replication by inhibiting topoisomerases or intercalating into DNA [36]. Many chemotherapeutics generate reactive oxygen species (ROS), with anthracyclines producing particularly high levels [37]. In contrast, the taxane docetaxel disrupts microtubules and generates comparatively low ROS [37, 38]. Consistent with this distinction, docetaxel sensitivity was not significantly reduced by metal exposure in our system.

Prolonged ROS exposure has previously been linked to chemoresistance, often through cell cycle arrest or impaired apoptosis [39]. Because heavy metals can increase ROS, we investigated whether this mechanism contributed to resistance. A subtoxic mixture of metals elevated ROS levels but did not markedly alter cell proliferation, suggesting that cells had not entered cell cycle arrest. These results suggest that metal-induced ROS contributes, at least partially, to reduced chemotherapy sensitivity, particularly for agents that rely on ROS generation, such as anthracyclines, alkylating agents, and camptothecins. Copper and manganese were the strongest contributors to doxorubicin resistance. Both metals are essential cofactors for ROS-scavenging enzymes such as superoxide dismutases [40], which may enhance cellular antioxidant capacity. Treatment with N-acetylcysteine (NAC) partially restored doxorubicin sensitivity.

Our work suggests that metal-induced chemoresistance is not simply driven by total ROS levels, but likely by source-specific, spatially restricted ROS generation coupled to metal-dependent signalling pathways. Transition metals such as copper generate ROS via Haber-Weiss or Fenton-like chemistry in the presence of H_2_O_2_ or O_2_^•-^, producing highly localised ROS species, especially at sites where these metals accumulate [41]. This contrasts with exogenous hydrogen peroxide, which acts as a diffusible oxidant and lacks spatial specificity, leading to acute cytotoxicity at higher doses [42]. Furthermore, metals such as manganese and zinc modulate antioxidant systems and redox-sensitive signalling independently of ROS levels, indicating that ROS and metal-dependent signalling cooperate to induce a chemoresistant phenotype. In contrast, menadione induces resistance through redox cycling and metabolic perturbations that we identified to be partly independent of bulk ROS accumulation, highlighting that ROS measurements alone do not fully capture the complexity of redox-mediated drug resistance. Future studies will focus on identifying the mechanisms that metals use to induce chemoresistance alongside ROS production.

Interestingly, oxaliplatin, despite being associated with ROS production, showed improved efficacy in the presence of metal ions. Oxaliplatin is a platinum-based agent used in several cancers, including lung cancer, and is transported into cells via Organic Cation Transporters (OCT1 and OCT2) [43, 44]. Previous reports indicate that cadmium can enhance oxaliplatin uptake through OCT2 [45], and lead can increase OCT2 expression in certain tissues [46]. These findings suggest that some metals may promote platinum drug uptake. Notably, because platinum agents such as cisplatin, oxaliplatin, and carboplatin rely on a platinum core [47], combining them with broad chelation strategies may not be advisable.

We next evaluated MiADMSA as a potential chelator to restore chemotherapy sensitivity. In metal-loaded cells, MiADMSA was the most effective of the chelators tested in reversing doxorubicin resistance. Spectroscopic UV–Vis titration demonstrated strong complex formation with copper, manganese, and lead. Binding analysis indicated moderate to strong affinity for zinc, cadmium, and lead (log K1 ~ 5.1–5.7), while manganese binding was weaker (log K1 ~ 4.3), consistent with its classification as a harder metal ion.

Although EDTA binds many metals with higher affinity, its lack of selectivity and strong binding to essential ions such as calcium and magnesium present safety concerns. Furthermore, EDTA is highly hydrophilic and poorly cell permeable. In contrast, MiADMSA contains an isoamyl group that increases lipophilicity, enabling intracellular access. In a closed cell culture system, complete removal is unlikely, but measurements of intracellular metal levels and metal reporter assays confirmed at least partial metal reduction after MiADMSA treatment. In addition, detection of a functional intracellular fluorinated analogue supports MiADMSA’s cell permeability.

Overall, our data demonstrate that combined metal accumulation promotes chemotherapy resistance in lung cancer cells. MiADMSA can enter cells, bind multiple metals, reduce intracellular metal burden, reduce ROS and restore drug sensitivity. Given that metal accumulation has also been reported in pancreatic, leukaemia, and colorectal cancers [48–50], further optimisation of this chelation strategy may have broader therapeutic potential.

## Method

### Cell lines

Beas-2B (origin: lung non-cancerous bronchial epithelium, male, ethnicity unknown, CRL-3588), A549 (origin: lung adenocarcinoma, male, Caucasian, CRM-CCL-185), and H1417 (origin: Small cell lung cancer, female, Caucasian, CRL-5869) cells were all obtained from ATCC (https://www.atcc.org). Adherent cells (Beas-2B & A549) were cultured in DMEM (Gibco, High glucose, L-Glutamine, pyruvate) supplemented with 10% Foetal bovine serum (FBS, Merck) at 37 °C under 5% CO_2_. Suspension H1417 cells were cultured in RPMI (Gibco) supplemented with 10% FBS under the same conditions. Cell lines were authenticated by Eurofins and PCR mycoplasma testing was carried out regularly to assess for infections.

### Animal work

All animal work was approved by the local AWERB committee and in accordance with the approved protocol. This work was licensed under PPL8383164 at the registered licence establishment X78C87E61. Animals were kept in environment-enriched IVC cages (3 per cage) with food and water ad libitum and randomly assigned to the study. Prior to tumour development, animals were exposed to metal mix for 6 weeks in total via oral gavage (3X per week). Animals were randomly allocated in groups of 3 consisting of; 1. Saline gavage, 2. Metal mix gavage, 3. saline gavage MiADMSA, 4. Metal mix gavage MiADMSA, 5. Metal mix gavage MiADMSA and doxorubicin. The metal mix contained Potassium dichromate (3.18 mg/kg), copper sulphate pentahydrate (7.86 mg/kg), Zinc sulphate heptahydrate (17.59 mg/kg), Cadmium chloride hemipentahydrate (0.51 mg/kg), lead acetate trihydrate (6.18 mg/kg), manganese chloride tetrahydrate (9.01 kg/mg) dissolved in water. After 6 weeks of metal exposure, mice were acclimatised for 1 week, after which 100 µl of 1 × 10^6^ A549 cells were injected subcutaneously. Once tumours on average reached 100 mm^3^, doxorubicin was administered intraperitoneally once per week for 2 weeks at 4 mg/kg, for 1 week at 2 mg/kg and 1 week at 1 mg/kg. One mouse in group 5 showed weight loss of more than 10% and some infection at the site of IP injection after the third week of doxorubicin treatment and was culled in week 4 as stipulated on our licence. As the study was reaching its end, all mice were subsequently culled with tumours reaching about 400 mm^3^. This was well under the maximum stipulated size of 1500 mm^3^ that was stipulated on our licence. Alongside doxorubicin, MiADMSA was co-administered intraperitoneally at 0.3 mMol/ kg. Tumours were measured with callipers in which volume was determined using the formula (width × length^2^)/2. Measurements were performed blindly.

### Patient data using online datasets

Xenabrowser was used [51] on the TCGA TARGET GTEx dataset to assess the gene expression of *MT2A* in cancer patients compared to controls in which gene expression was divided in quartiles. Samples without data were excluded. Data from the TCGA Lung cancer dataset was extracted to generate Kaplan–Meyer survival curves using GraphPad Prism using only primary tumours.

### Metals, chemotherapy and chelators

A mixture of six metals, three endogenous and three exogenous, were combined as indicated in Table 1 to generate a solution that could be standardised and given to cells in a certain dilution. Metal salts were solubilised into 1 M grandparent solutions in 100 mM HNO_3_ before being diluted further in distilled autoclaved water into parent solutions at 10 mM.

As metal salts were dissolved in acid (65% HNO_3_, Merck) to prevent oxidation_,_ NaOH (Merck) was added in equal concentrations and volumes to neutralise the media and maintain a stable pH. All controls are therefore treated with an acid compensation media, consisting of equimolar amounts of NaOH and HNO_3_ at amounts used when exposing to metals.

Chelators EDTA (Thermo Fisher Scientific), DMPS (Merck)and DMSA (Merck) were dissolved in water. Chemotherapeutics were made into stock solutions and then diluted as required for each titration curve. Doxorubicin (Merck) stock 10 mM in water, Etoposide (Merck) stock 50 mM in DMSO, Gemcitabine hydrochloride (Merck) stock 33.4 mM in water, docetaxel (Merck) stock 6.2 mM in DMSO, oxaliplatin (Merck) stock 12.6 mM in water.

Chelator MiADMSA (patent application WO2006033907A2) and analogues of this chelator are dissolved in a nitrogen saturated 5% sodium bicarbonate (Merck) solution and immediately used.

### Constructs and transfections

Constructs were amplified by transforming into competent Dh5alpha *E. coli* and purified using a GeneJET Plasmid Midiprep Kit (Thermo Fisher Scientific). A nanodrop spectrophotometer (LabTech International) was used to measure DNA concentration and purity. Plasmids were stored at 4 °C for short term or −20 °C for longer term.

Unless otherwise stated all transfections were performed with polyethyleneimine (PEI 25 K, Polysciences) at a concentration of 1 mg/ml in water. 3 µl PEI and 1 µg plasmid were combined in 250 µl of serum free media, vortexed and incubated for 15 min at RT. The transfection mixture was then added dropwise to cells cultured in 6-well plates. Media was refreshed 6 h post transfection.

TK Renilla and MRE luciferase were used as in previous publications [52, 53].

### Luciferase assays

Adherent cells were seeded into 6-well plates, before each well was co-transfected with 600 ng metal response element (MRE) luciferase and 60 ng TK Renilla, using PEI. Six hours post transfection, cells were trypisinised and split into 96 well plates, 10,000 cells per well for treatment. Metals were added 24 h post transfection, followed by chelators 6 h after metal exposure. Cells were then incubated for a further 24 h before harvest for luciferase measurements.

Cells were washed in PBS twice before lysis in 1X passive lysis buffer (Promega) for 15 min at RT. 10 µl aliquots of lysate from each well was then transferred into an opaque white 96 well plate (Sarstedt) before luciferin reagent (Promega) and stop-and-glo Renilla substrate (Promega) were sequentially added to each well and luminescence read using the Dual Luciferase Reporter Assay system (Promega) in the GloMax 96 dual injector Microplate Luminometer (Promega, Southampton, UK). Firefly luciferase measurements were corrected for Renilla luciferase values and are displayed as relative luminescence units (RLU).

### Cell survival assays

For adherent cell lines, 1000 cells per well were seeded into a 96 well plate. 24 h after seeding, media was removed, and cells were treated with 90 μl of mixed metal solution or acid compensation medium as a control for 24 h. Chemotherapeutic agents with or without chelators and NAC (Merck) were then added for a further 72 h. Most typically this would be doxorubicin (Sigma-Aldrich) at increasing concentrations up to 2.4 μM, and chelators at a concentration of 18.75 μM for MiADMSA (Pleco Therapeutics) or 150 μM for other chelators as indicated in figure legends. 10 μl of these compounds were added directly on to the metal-loaded cells. After 72 h of incubation, media was replaced with a 44 μM resazurin solution (Sigma Aldrich, Merck). The cells were incubated for up to 4 h and resorufin was detected using a GloMax 96 Microplate Luminometer at excitation of 570 nm and emission of 583 nm (Promega, Southampton, UK). For the H1417 suspension cells, metal solutions and compensation media are added at the time of seeding to avoid aspirating viable cells after 24 h.

For analyses, data was converted to a percentage of the control value (0 chemotherapy/0 metal) for each condition and plotted to form a sigmoidal dose-response curve using GraphPad Prism version 11. Viability of these conditions was recorded and displayed in supplemental data. The IC_50_ or IC_25_ values for each condition were derived from the dose-response curves.

### ICP-MS

Cells were seeded into 10 cm plates for 24 h and treated as required, with mixed metal solutions and chelators or other treatment compounds as indicated. Once confluent, cells were washed twice in PBS and lysed on ice in 150 μl of NP40 lysis buffer (100 mM NaCl, 100 mM Tris-Cl pH 8, 1% NP-40).100 μl of this lysate was then dried in a vacuum dehydrator (Eppendorf 5301 SpeedVac) before dried samples were dissolved in 100 μl of concentrated HNO_3_ (Merck) and further processed by heating at 65 °C for 30 min, followed by boiling at 99 °C for 30 min. These samples were then diluted 50-fold in 2.5% HNO_3_ containing 10 mg/ml internal standards (In, Ag, Be) for ICP-MS analysis.

Digested and diluted ICP-MS samples were run in the iCAP RQ QCell CRC ICP-MS (Thermo Fisher Scientific) on KED mode using pure He gas to quantify concentrations of cadmium, chromium, lead, manganese, copper and zinc ions. Calibration curves were produced for each quantified metal using standard solutions, purchased from CertiPrep Claritas and Fisher scientific.

The remaining 50 µl lysate was used for protein determination. A Pierce BCA assay (Thermo Fisher Scientific) was performed on the lysate, with two replicates of 25 μl each in 200 μl of working solution diluted as described in the manufacturer’s protocol. Each set of duplicate samples was also run with eight standards with known protein concentrations and a NP-40 lysis buffer blank. ICP-MS data for cultured cells was normalised relative to the protein concentration.

### Fluorescent microscopy

Beas-2B cells were seeded on glass coverslips and treated with a combination of 1X metals and 18.75 µM MiADMSA for 48 and 24 h respectively. Doxorubicin at 2 and 8 µM was added 1 h prior to fixation. Treated cells were then washed once with PBS and fixed with 4% PFA at RT for 10 min. The coverslips were mounted onto glass slides with mounting fluid (Thermo Fisher Scientific). The fluorescent images were obtained with a Zeiss Apotome (Zeiss, Cambridge) keeping the exposure between images the same (38.682 ms). 3 × 100 cells per condition were analysed for fluorescent intensity per particle (cell) using Fiji software.

### RT-qPCR

Beas-2B cells were cultured 1:8 in 6-well plates. RNA was extracted from cells using the Norgen Biotek Total RNA Purification Kit, RNA concentrations were quantified using a nanodrop spectrophotometer (LabTech International). 500 ng RNA was then synthesised into cDNA with the Applied Biosystems cDNA Reverse Transcription Kit, according to the manufacturers protocol. 1:50 dilutions of cDNA were then made and stored at –20 °C. RT-qPCR plates were prepared using the PowerUp SYBR Green Master Mix (Applied Biosystems) with 0.5 μM of primers, outlined in Supplementary Table S4. RT-qPCR reactions were carried out in CFX Duet Real Time PCR system, using the following cycle: 40 cycles of Denaturation (95 °C, 15 s), annealing (60 °C, 60 s), extension (60 °C, 60 s), and a final storage step at 4 °C. Negative controls, including those with no reverse transcriptase and no RNA were included in this study. Expression of genes was normalised against GAPDH which was used as the reference gene in these experiments.

### ROS assays

Beas-2B cells were cultured in a 96 well plate for 24 h prior to treatment (10,000 cells per well, 80 µl per well). Cells are treated with metals, chelators, NAC (Sigma), Menadione (Merck) and H_2_O_2_ (Merck) for 24 h. 6 h prior to harvest 20 µl of diluted H_2_O_2_ substrate solution (Promega ROS-Glo kit) is added to each well. After incubation with substrate, 50 µl of media from each well is transferred into an opaque white 96 well plate and combined with 50 µl of ROS-Glo Detection Solution (Luciferin detection solution, signal enhancer, D-cystine, Promega) before a further incubation of 20 min. Luminescence is then detected in a Promega Glo-Max plate reader. Cells are then lysed and a Pierce BCA assay (Thermo Fisher Scientific) performed to normalise the luminescence value by protein content.

### UV–Vis spectroscopy metal titrations

Ultraviolet-Visible (UV–Vis) spectroscopy spectra were recorded on a Cary 5000 UV–Vis–NIR spectrophotometer, using a 10 mm path length quartz glass Hellma semi micro cell cuvette, scanning in the 200–600 nm wavelength range.

Solutions of MiADMSA were prepared in 0.1 M HEPES at pH 7.4 and used immediately after dissolving (1 mM diluted from a 10 mM stock solution of 25.5 mg MiADMSA in 10 mL HEPES buffer). All metal solutions were prepared in 10 mM HNO_3_, using the following divalent metal salts: CuSO_4_·5H_2_O, ZnCl_2_, MnCl_2_·4H_2_O, CdCl_2_·2.5H_2_O, Pb(OAc)_2_·3H_2_O. Aliquots of metal solution (5 μL) were titrated into a sample cuvette containing 1 mL of 1 mM MiADMSA in increments of 0.05 equivalents relative to MiADMSA, with the cuvette shaken and left for one minute before recording a spectrum. An equal volume of 10 mM HNO_3_ was added into the reference cuvette (containing 0.1 M HEPES at pH 7.4) addition of metal solution to the sample cuvette to account for the effects of sample dilution and evolving solvent matrix.

### Estimation of metal binding constants

Estimations of binding constants for the formation of MiADMSA metal complexes were obtained by fitting multi-wavelength absorbance data from UV-Vis titrations to a 2:1 L-BFGS-B model using BindFit v0.5 (https://supramolecular.org). These values are presented in the supporting information (Supplementary Table S3).

### NMR analysis

All starting materials and reagents were purchased from commercial sources and used as received. All reactions were conducted under an atmosphere of air. Column chromatography was carried out on silica purchased from Fluorochem using hexane/ethyl acetate solvent systems or using a Combiflash Nextgen 100 equipped with a redisep column using hexane/ethyl acetate solvent systems.

^1^H NMR spectra were recorded at 400 or 600 MHz using Bruker Avance III or Varian VNMRS-600 spectrometers respectively. ^13^C NMR spectra were recorded at 100 or 151 MHz using a Bruker Avance III or Varian VNMRS-600 respectively. ^19^F NMR spectra were recorded at 376 MHz using a Bruker Avance III spectrometer. All coupling constants are reported in Hertz (Hz). In cases where it was required 2D NMR techniques were used to confirm compound identity. Chemical shifts are reported in ppm and are referenced to residual solvent peaks; CHCl_3_ (^1^H 7.26 ppm, ^13^C 77.0 ppm), CH_3_CN (^1^H 1.94 ppm, ^13^C ppm) or DMSO (^1^H 2.50 ppm, ^13^C 39.5 ppm). Diastereoisomer ratios were determined through integration of relevant signals in ^1^H NMR spectra.

Mass spectra were collected either using ESI-LC or GCMS. ESI-LC in MeCN were collected using a Waters TQD mass spectrometer with a Acquity UPLC BEH C18 1.7 µm (2.1 mm × 50 mm). ESI-LC was collected using water containing formic acid (0.1% v/v) and MeCN mixture in a 95:5 to 5:95 gradient over 5 min. GCMS experiments were carried out on a Shimadzu QP2010-Ultra with a Rxi-5Sil MS (0.15 μm × 10 m × 0.15 mm). Helium was employed as the carrier gas (0.41 mL/min). EI is carried at 70ev and the working mass range is 35–650 u for all GCMS experiments.

ASAP samples were run isothermally at 350 °C vaporising the sample to enable atmospheric pressure chemical ionisation.

^19^F experiments were collected using a 700 MHz Bruker spectrometer equipped with a BBO CryoProbe. No decoupling was used. The spectral width spanned from –165.5 ppm to 33.8 pp. The number of points was 250,000. The repetition time was 2.95 s of which 0.95 s included the acquisition. The experimental time was 1 day and 12 min and it required 30448 transients (scans). The fluorine background produced by probe components was attenuated using depth pulses [54] as implemented by Bruker, but a flat baseline could only be achieved using Bruker’s apbks algorithm after the depth pulses. Please see supplemental methods for the background suppression pulse scheme. We note that the apbks algorithm could have been enough. All samples were dissolved in methanol-d_4_.

### MiADMSA CF_3_ analogue- synthesis of 2,3-bis(acetylthio)-4-oxo-4-((4-(trifluoromethyl)benzyl)oxy)butanoic acid

*S*,*S*’-((3 *R*,4*S*)-2,5-dioxotetrahydrofuran-3,4-diyl) diethanethioate (0.80 g, 3.22 mmol) was dissolved in toluene (10 mL) and 4-(trifluoromethyl) benzyl alcohol added (0.49 mL, 3.54 mmol). The solution was left to stir at 40 °C for 5 d before being concentrated under reduced pressure and purified by silica gel flash column chromatography (hexane : ethyl acetate, 1 : 0 to 1 : 1), affording the product as a clear oil (0.42 g, 0.98 mmol, 31%) with a diastereomeric excess of ≥95%.

^1^H NMR (599 MHz, CDCl_3_) δ 7.61 (d, *J* = 8.1, 2H), 7.41 (d, *J* = 8.0, 2H), 5.22 (s, 2H), 5.00 (d, *J* = 4.3, 1H), 4.98 (d, *J* = 4.4, 1H), 2.39 (s, 3H), 2.39 (s, 3H).

^19^F NMR (376 MHz, CDCl_3_) δ -62.60.

^13^C NMR (151 MHz, CDCl_3_) δ 192.74, 192.56, 175.06, 169.16, 139.04, 130.61 (q, *J* = 32.5), 128.04, 125.70 (q, *J* = 3.8), 124.09 (q, *J* = 272.2), 67.01, 46.55, 46.45, 29.99, 29.90.

HRMS (ESI) *m/z* calculated for [M + H]^+^ C_16_H_16_F_3_O_6_S_2_^+^ 425.0340, found 425.0342.

### MiADMSA CF_3_ analogue- synthesis of *anti*-2,3-dimercapto-4-oxo-4-((4(trifluoromethyl)benzyl)oxy)butanoic acid

2,3-bis(acetylthio)-4-oxo-4-((4-(trifluoromethyl)benzyl)oxy)butanoic acid (0.32 g, 0.75 mmol) was placed under an atmosphere of argon and dissolved in a pre-sparged (argon) solution of PBS buffer (0.01 M, pH 7.5–8.5, 8 mL), MeOH (3.2 mL) and thioglycolic acid (0.31 mL, 4.50 mmol). The solution was left to stir at 40 °C under argon for 72 h after this time the solution was heated to 50 °C for an additional 48 h before being heated to 60 °C for a further 24 h. The resulting white precipitate was filtered under reduced pressure with washings of water and hexane, affording the product as a white solid (75.8 mg, 0.22 mmol, 30%).

^1^H NMR (599 MHz, MeOD) δ 7.67 (d, *J* = 8.1, 2H), 7.61 (d, *J* = 8.1, 2H), 5.31 (s, 2H), 3.71 (d, *J* = 10.7, 1H), 3.59 (d, *J* = 10.7, 1H).

^19^F NMR (376 MHz, MeOD) δ −64.11.

^13^C NMR (151 MHz, MeOD) δ 173.86, 172.18, 141.52 (d, *J* = 1.5), 131.31 (q, *J* = 32.3), 129.48, 126.42 (q, *J* = 3.9), 125.58 (q, *J* = 271.2), 67.25, 45.74, 45.67.

HRMS (ESI) *m/z* calculated for [M-H]^-^ C_12_H_10_O_4_F_3_S_2_^+^ 338.9973, found 338.9973.

### Statistics

All data is presented, and statistics were done using GraphPad Prism 11. Samples were assumed to follow a Gaussian (normal) distribution as for most *n* = 3 studies is done. Unless otherwise indicated, one-way Anova with post-hoc Tukey test were used for testing of multiple comparisons, whereas for comparisons between two samples, parametric T-tests were used. Previous experiments suggested that 3 biological replicates were sufficient for most of the experiments. Any deviations of this are annotated in the legends. Each data point represents the mean of three replicates from a minimum of three independent repeats plotted with standard error. *p < 0.05, **p < 0.01, ***p < 0.001, ****p < 0.0001.

## Materials availability

The raw data supporting this research will not be published for commercial reasons.

## Supplementary information

Supplemental material

Supplemental Figures
