# Supplementary material for: Multi-metal cooperation drives chemoresistance in lung cancer and is reversed by the membrane-permeable chelator MiADMSA
Source: Cell Death Discov. 2026 Jul 6;12:339. doi: 10.1038/s41420-026-03222-8 (PMC13486686; doi:10.1038/s41420-026-03222-8)

Supplemental Figure 1

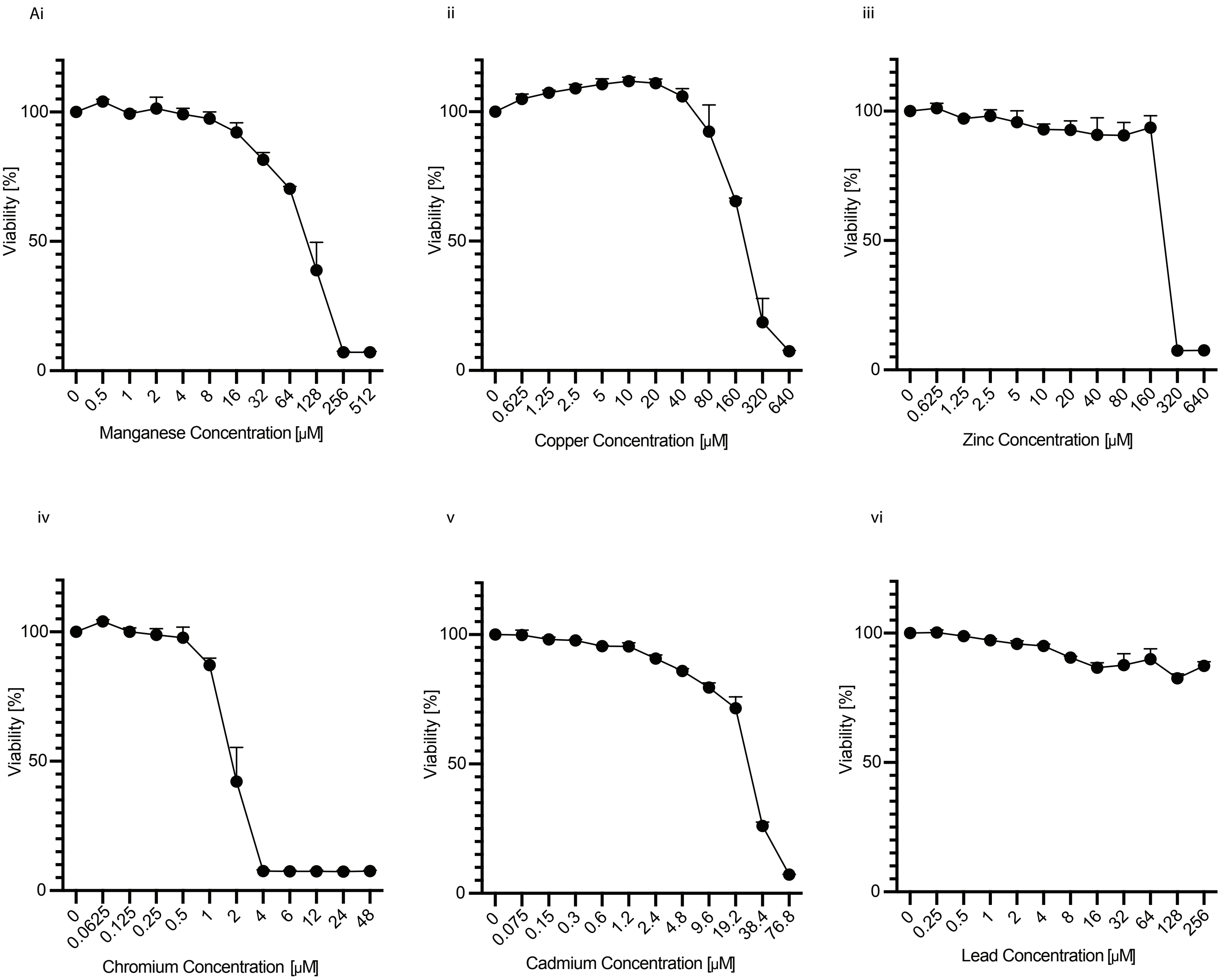

Supplemental Figure 2

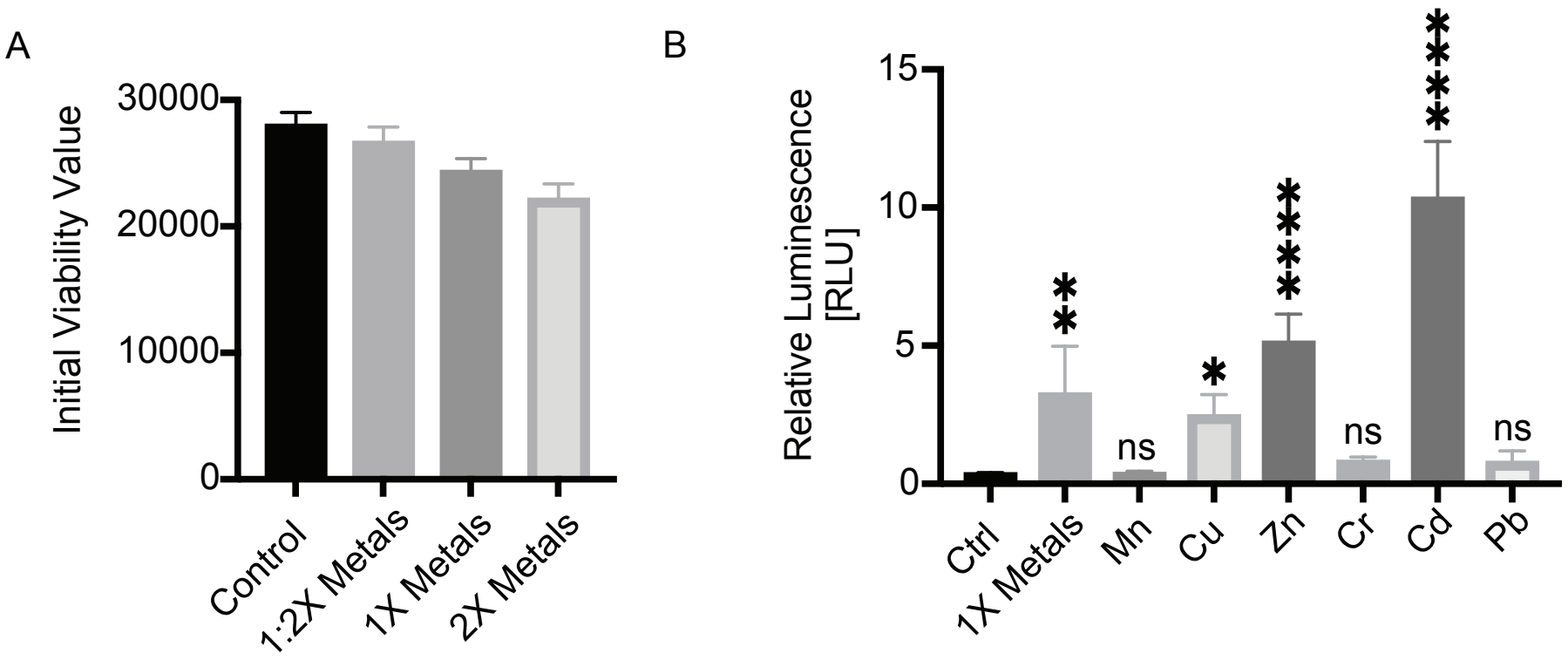

Supplemental Figure 3

A549 Cells

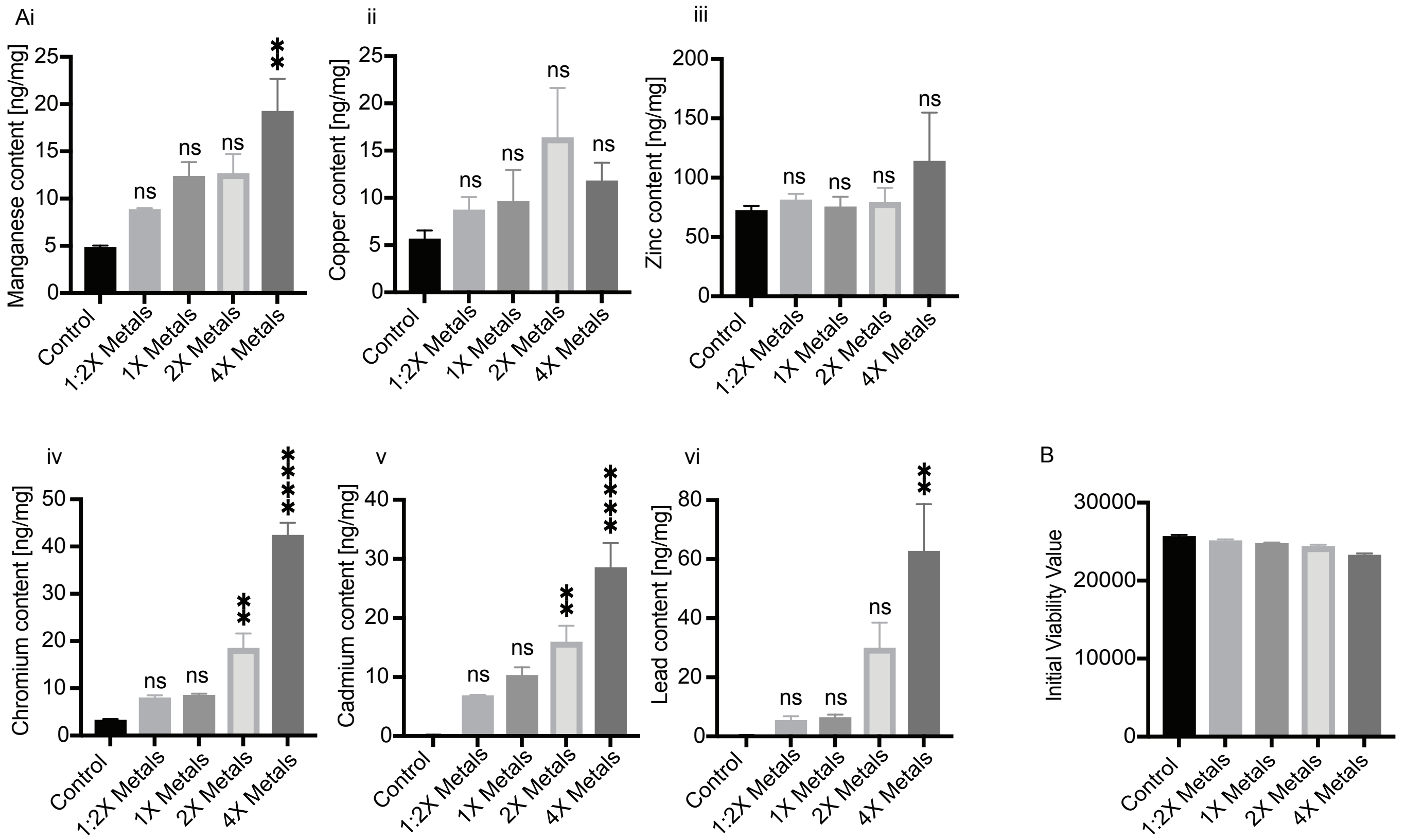

H1417 Cells

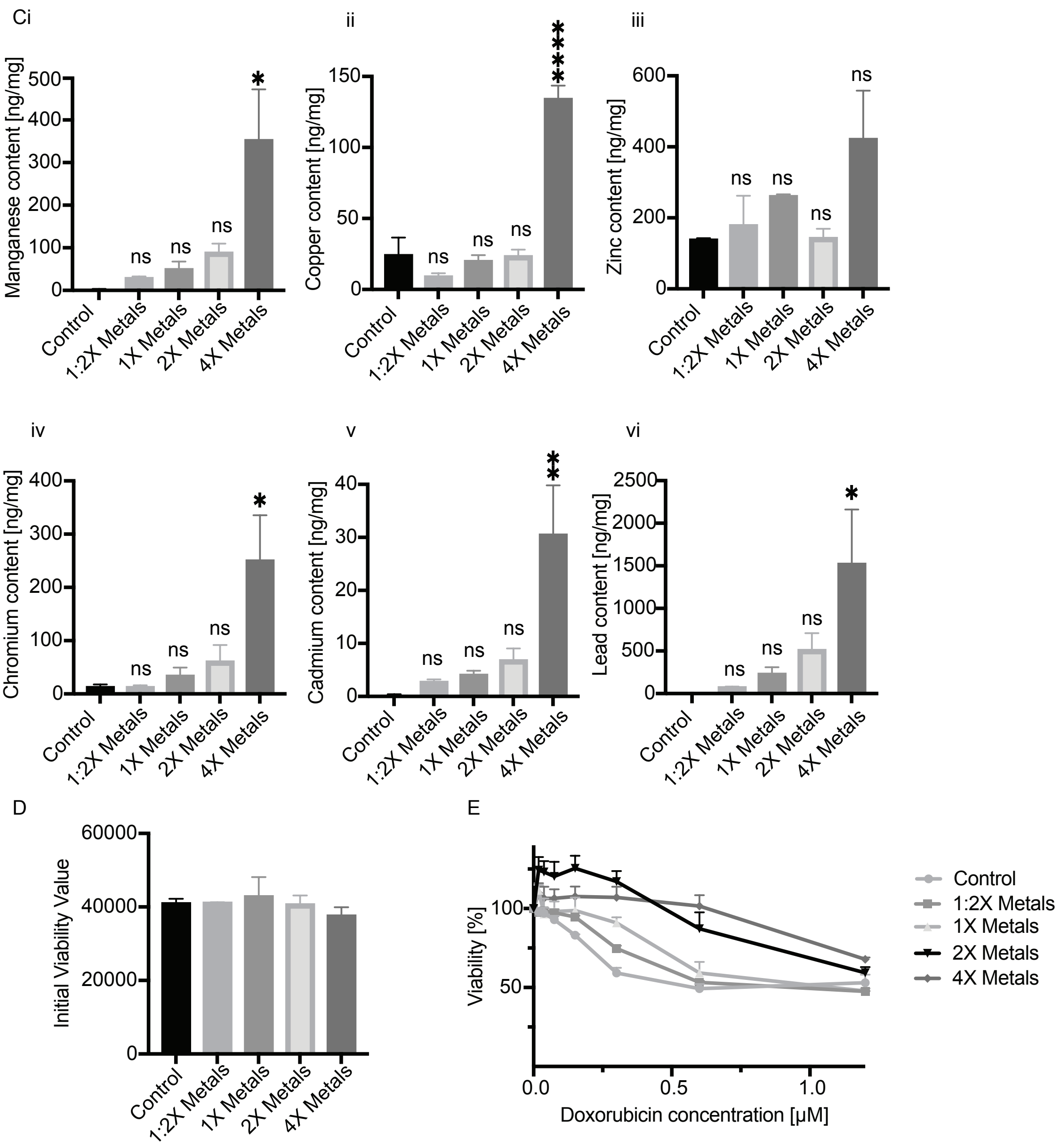

Supplemental Figure 4

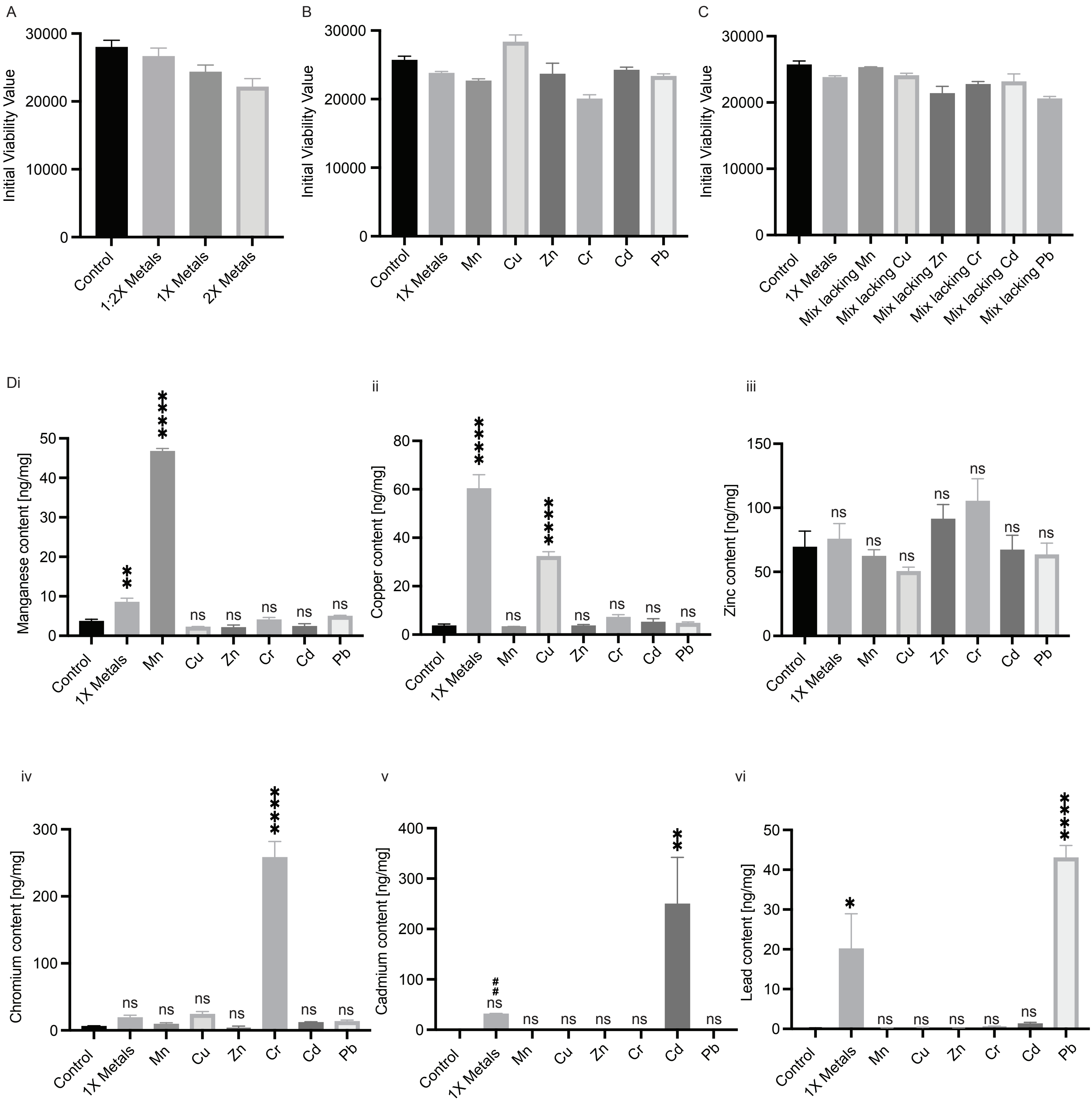

Supplemental Figure 5

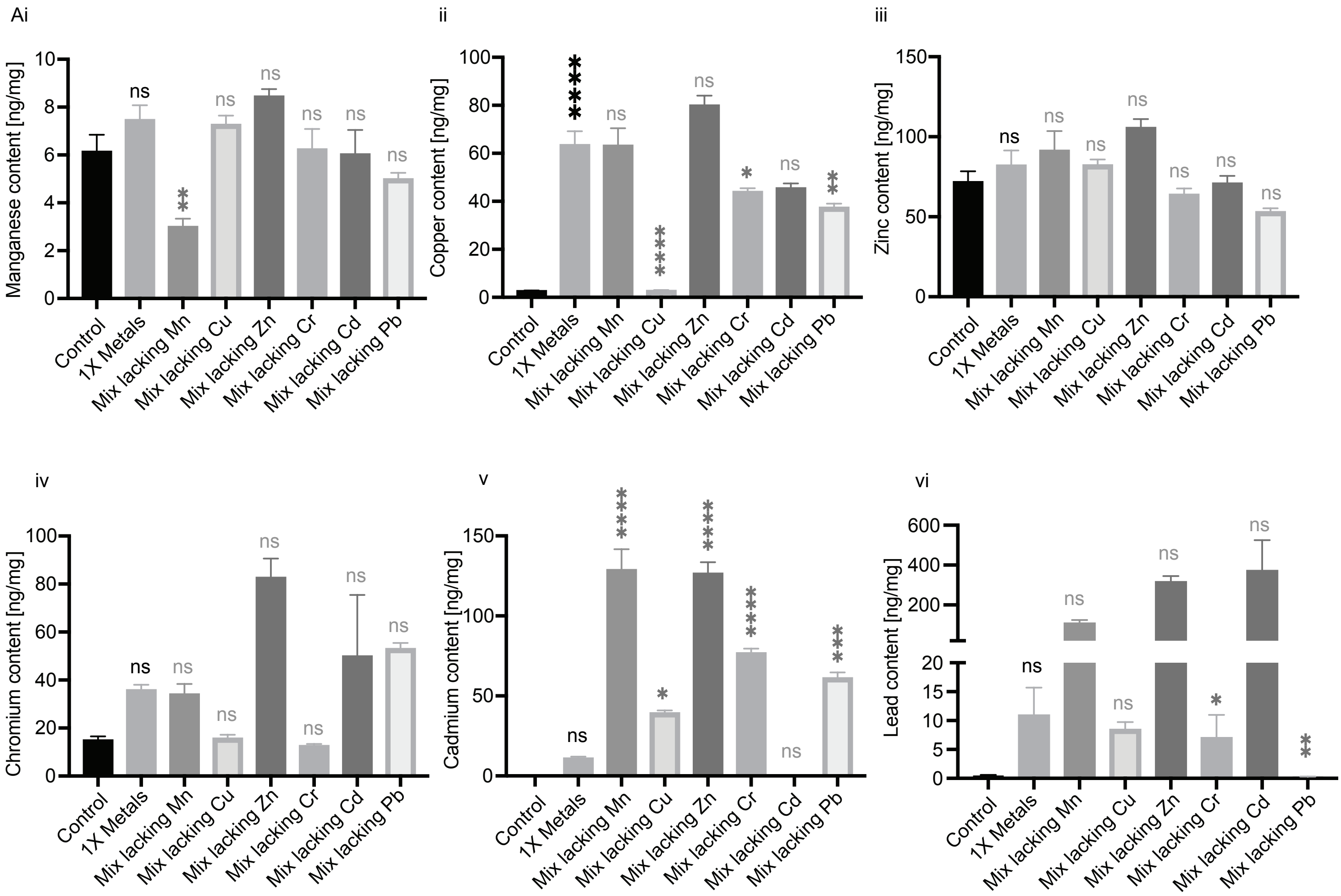

Supplemental Figure 6

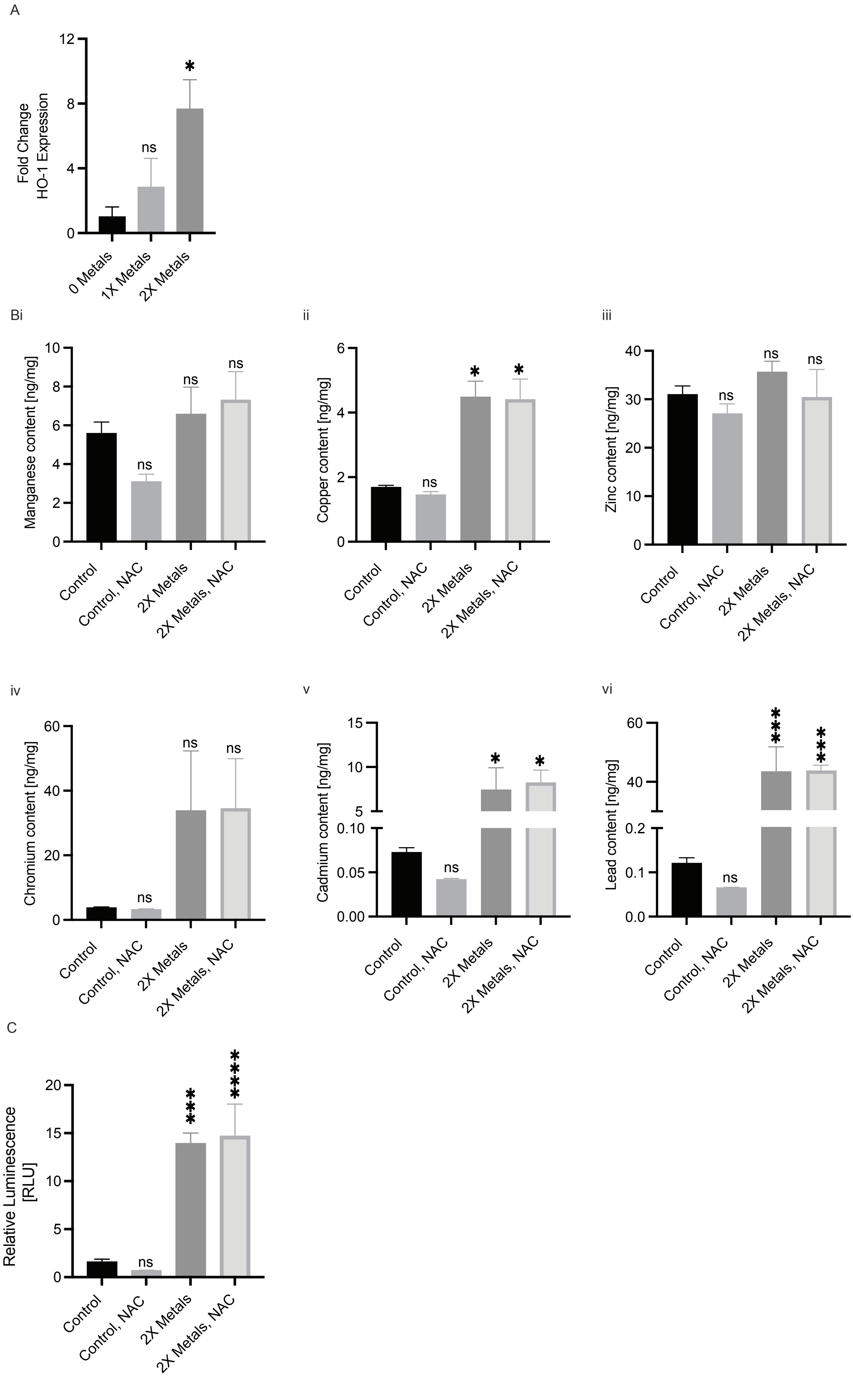

Supplemental Figure 7

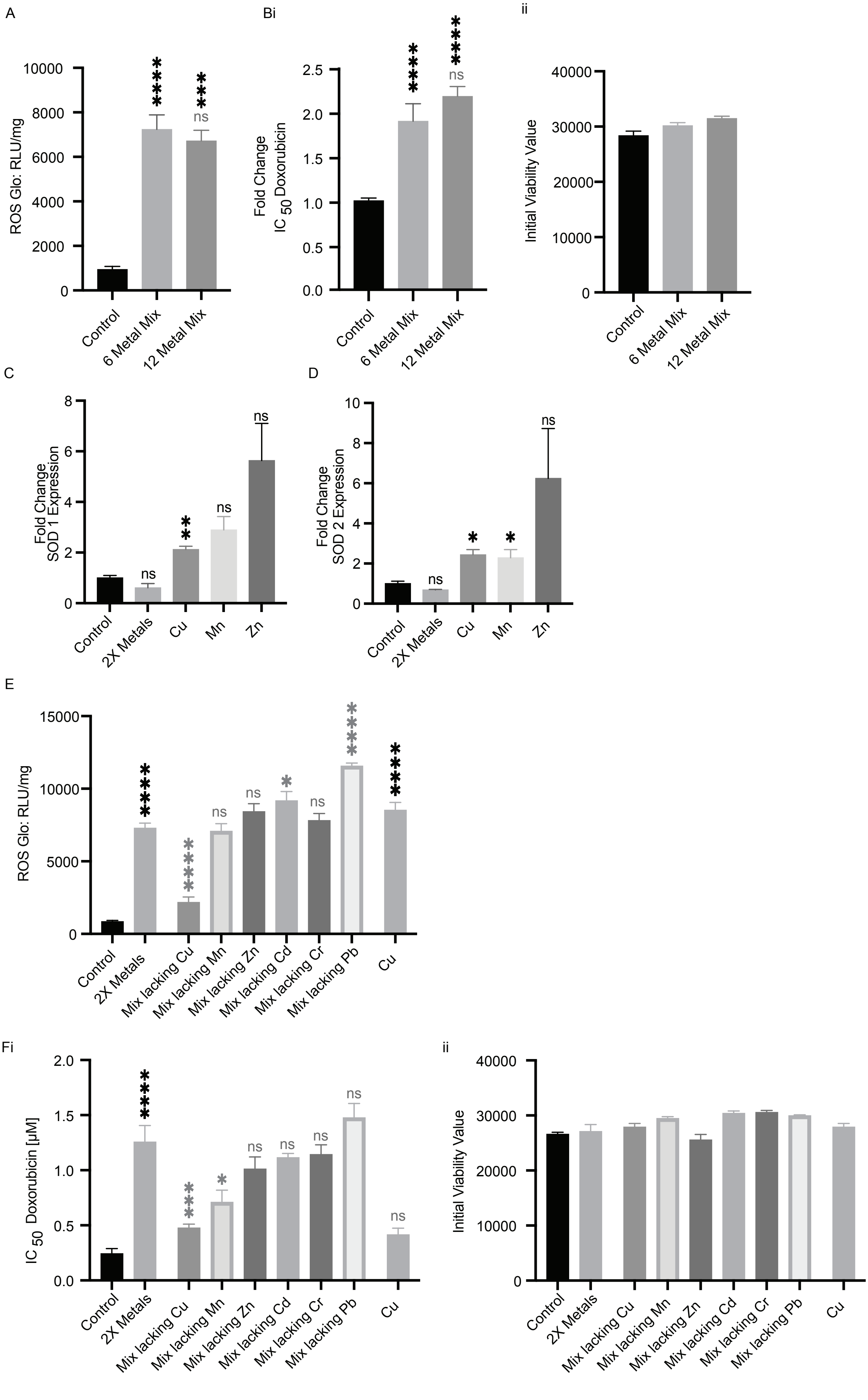

Supplemental Figure 8

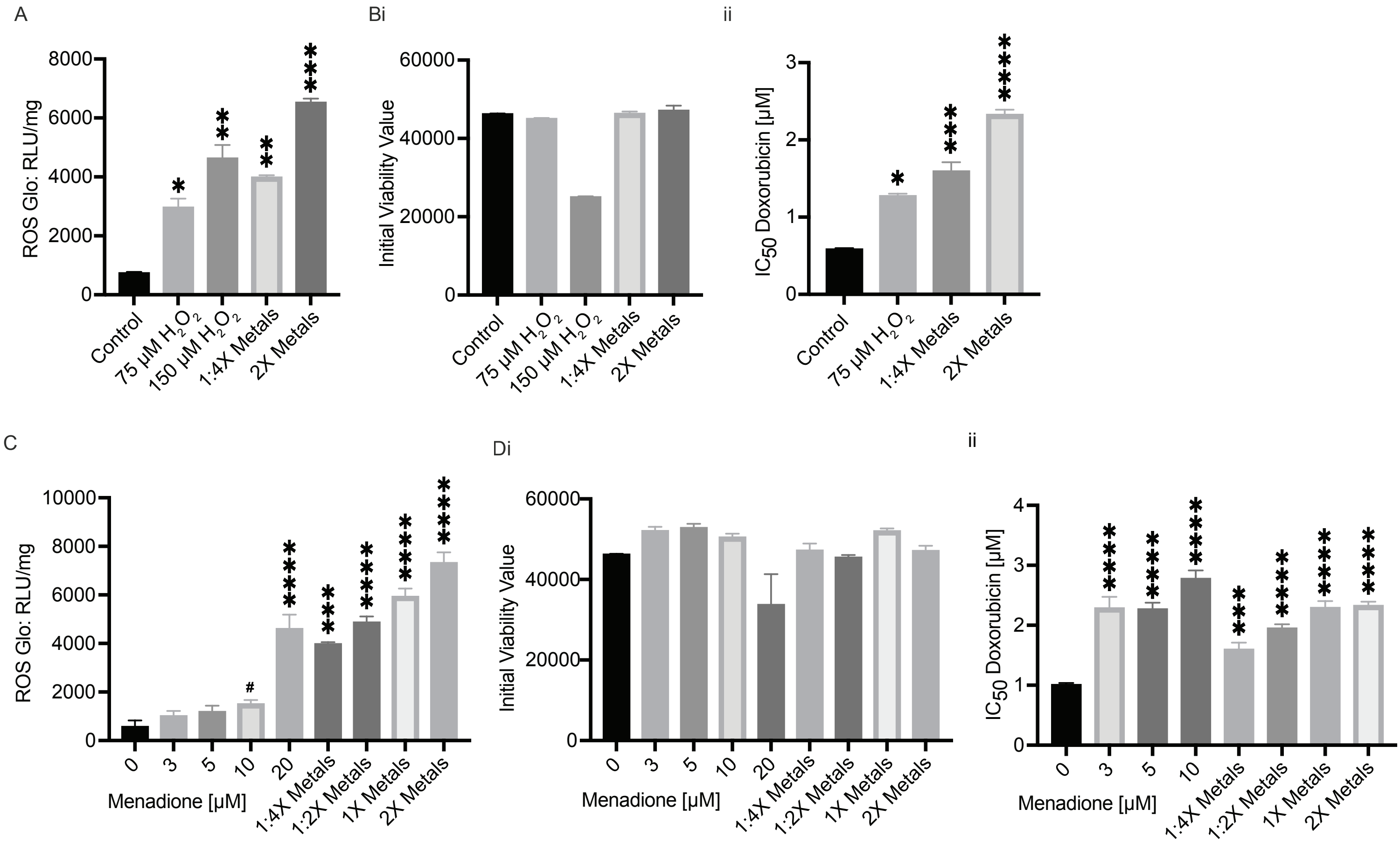

Supplemental Figure 9

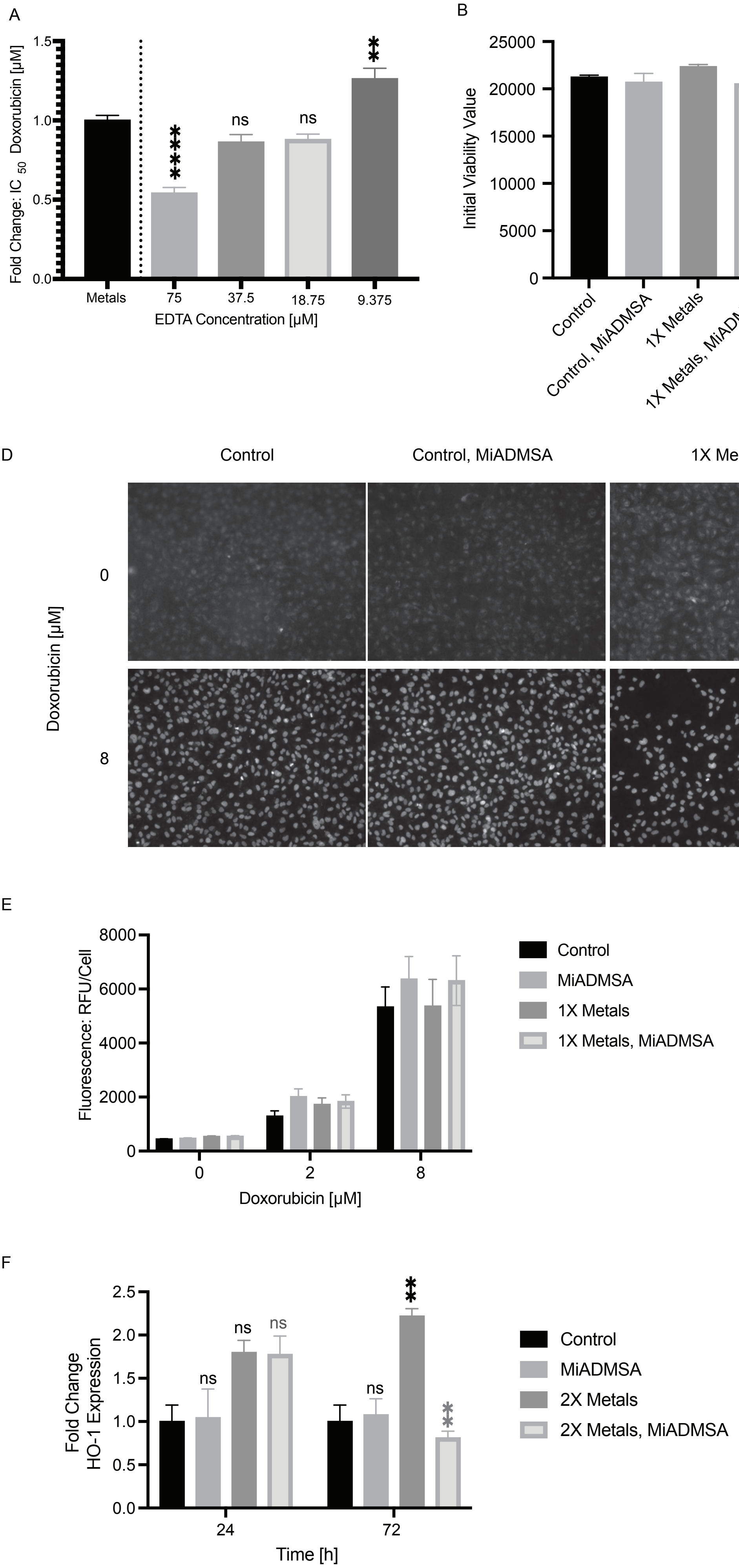

Supplemental Figure 10

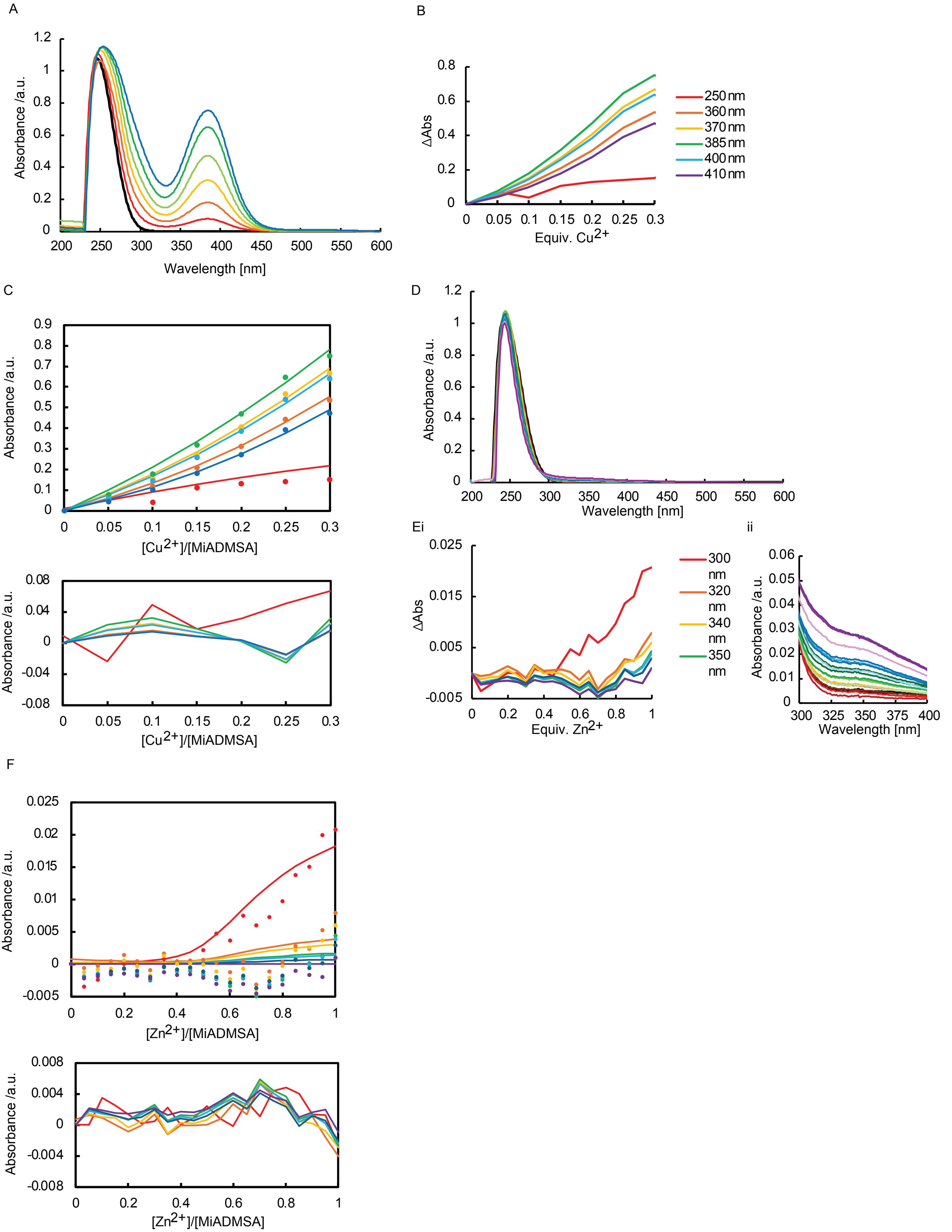

Supplemental Figure 11

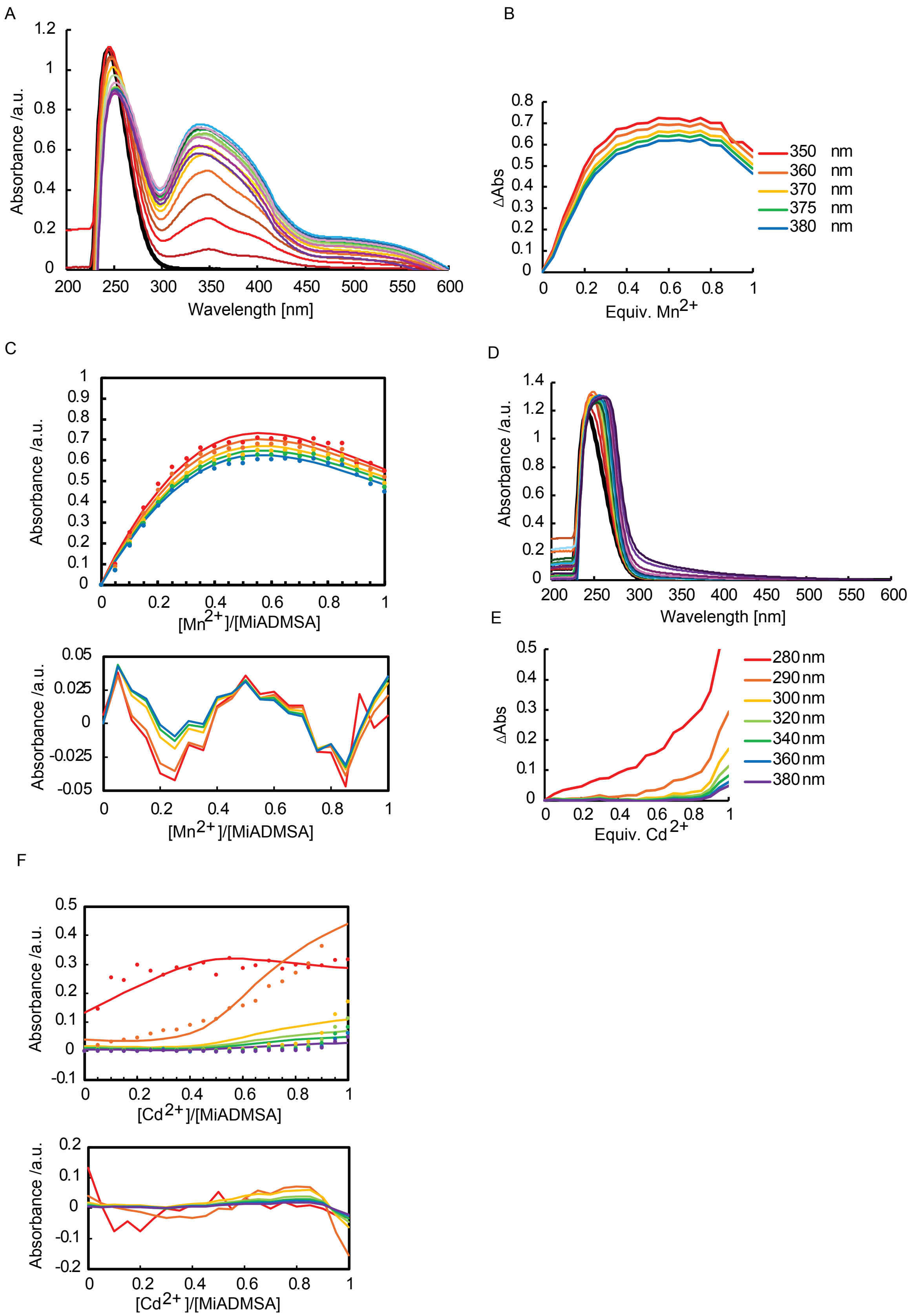

Supplemental Figure 12

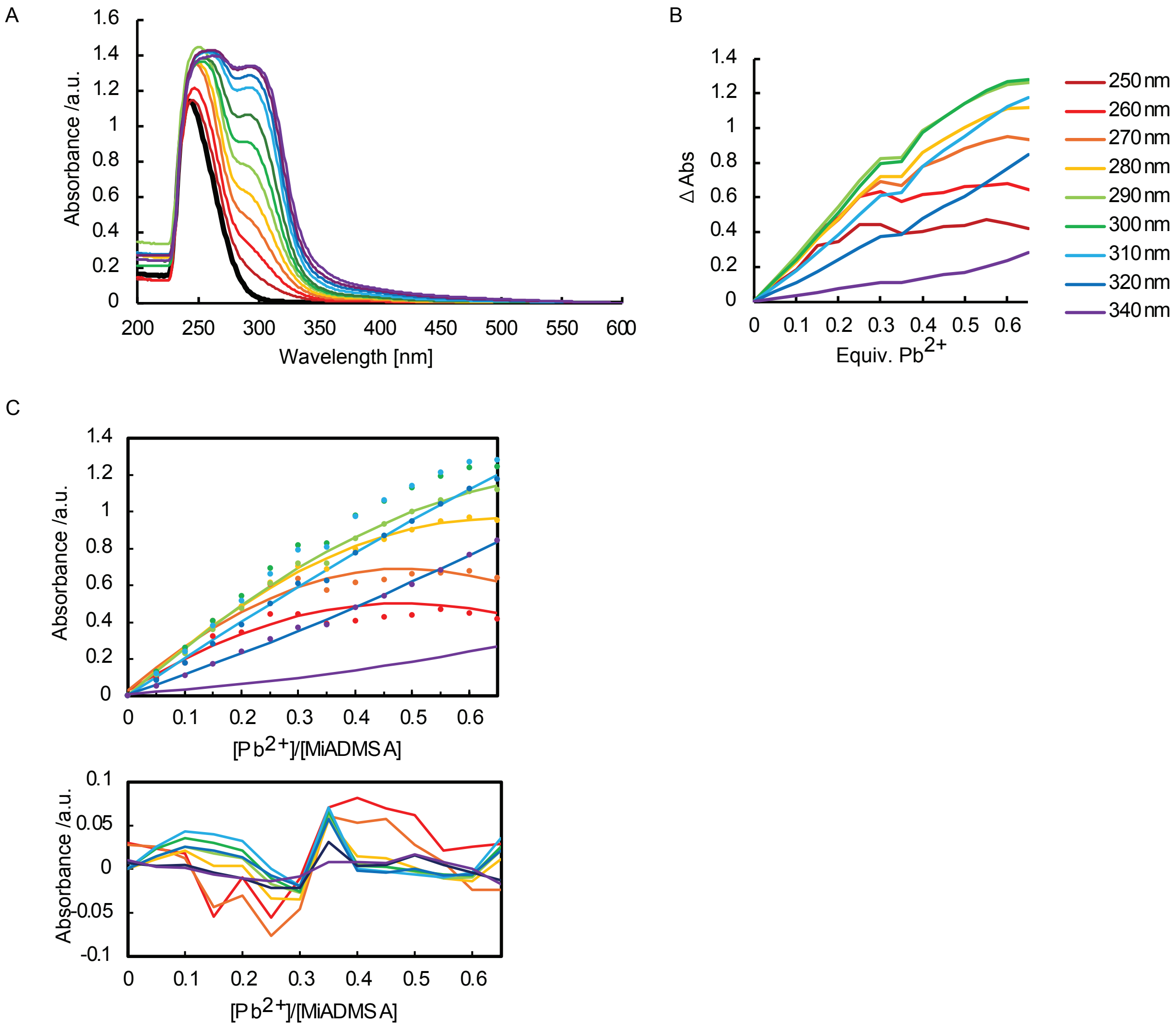

Supplemental Figure 13

A549 Cells

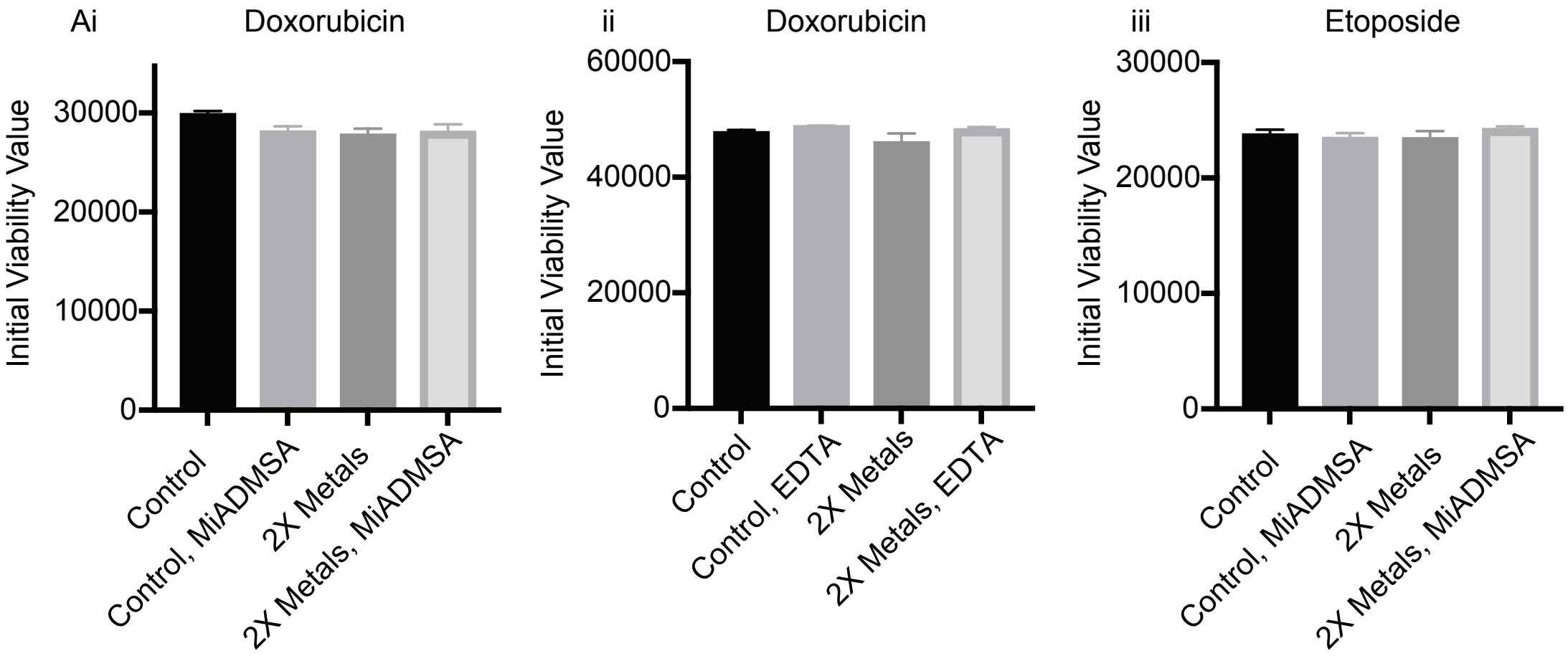

H1417 Cells

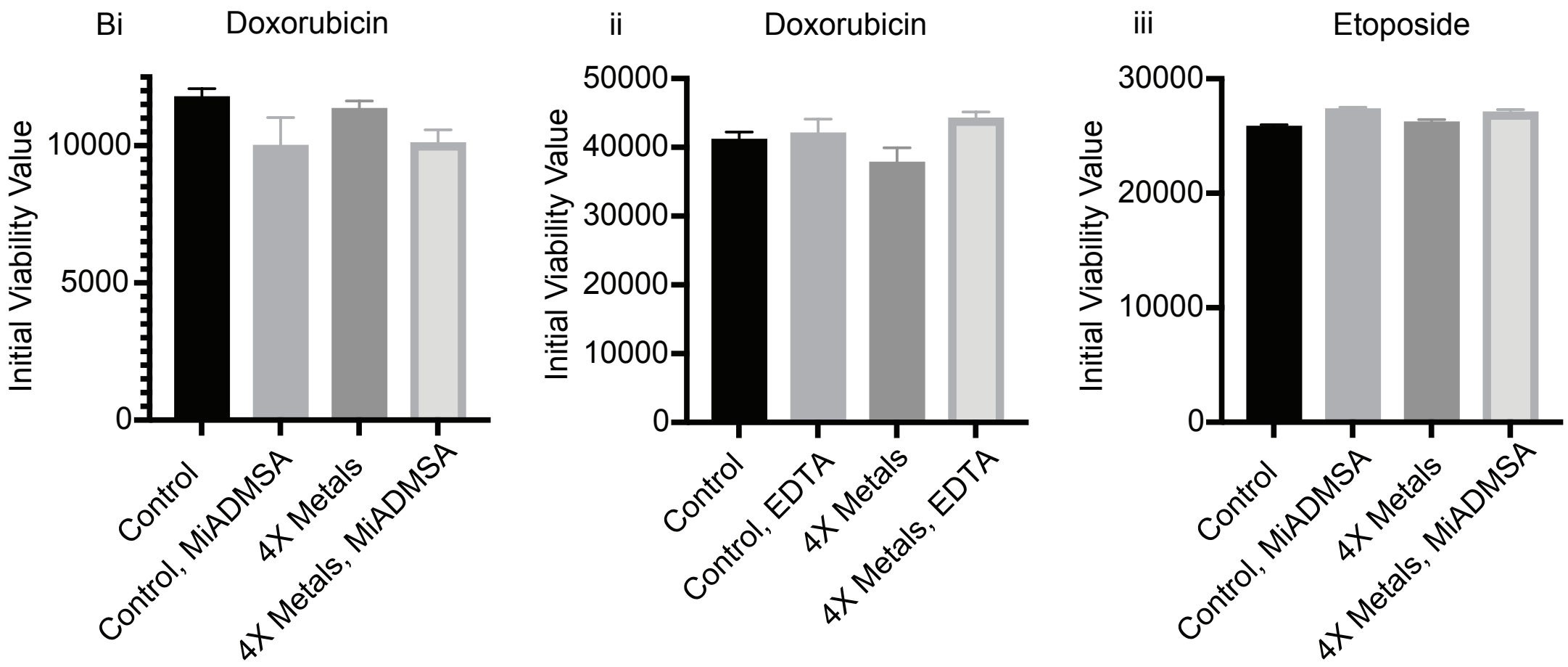

Supplement: Supplementary file 1 [file 41420_2026_3222_MOESM2_ESM.pdf]
