# Supplementary material for: Multi-metal cooperation drives chemoresistance in lung cancer and is reversed by the membrane-permeable chelator MiADMSA
Source: Cell Death Discov. 2026 Jul 6;12:339. doi: 10.1038/s41420-026-03222-8 (PMC13486686; doi:10.1038/s41420-026-03222-8)
Supplement: Supplementary file 2 [file 41420_2026_3222_MOESM1_ESM.docx]

**Supplemental Tables**

**Supplemental Table S1 literature review of metals detected in lung cancer patients**

|  | Fold change of metal in lung cancer when compared to control | | | | | | | | | | | | | |  | |
| --- | --- | --- | --- | --- | --- | --- | --- | --- | --- | --- | --- | --- | --- | --- | --- | --- |
| Ref | ^55^ | | ^56^ | | ^57^ | | ^58^ | | ^59^ | | ^60^ | | ^20^ | | ^61^ | |
|  | S | T | S | T | S | T | S | T | S | T | S | T | S | T | S | T |
| Cu | 1.4 | 1.62 | - | 1.09 | - | - | 13.7 | - | 0.56 | - | - | 1.19 | - | - | 1 | 1.3 |
| Zn | 0.86 | 0.66 | - | 0.92 | - | - | 22.5 | - | 0.26 | - | - | - | - | - | 1 | 1.4 |
| Mn | - | - | - | - | - | - | - | - | 30 | - | - | 0.69 | - | up | - | - |
| Pb | - | - | - | 0.88 | - | 0.59 | 32 | - | 5.2 | - | - | 1.79 | - | up | - | - |
| Cd | - | - | - | - | - | 2.24 | 20 | - | 1.4 | - | - | 0.78 | - | - | - | - |
| Cr | - | - | - | 1.01 | - | - | 34.3 | - | - | - | - | - | - | up | - | - |

Table to highlight which metals have been identified in lung cancer patient tumour tissue (T) or serum (S). Numbers indicate the fold increase in serum samples in lung cancer patients compared to controls or in tumour samples in lung cancer patients compared to controls. * In this manuscript only a percentage of patients that had increased metal levels rather than actual metal levels and therefore no number could be calculated. – means that the metal was not presented in that manuscript. Grey means the metal is not considered to change significantly compared to the control.

**Supplemental Table S2: IC50 and IC_10_ values for each metal** IC50 and IC10 values for metal survival in Beas-2B cells

| Metal | IC_50_ Value (µM) | IC_10_ Value (µM) |
| --- | --- | --- |
| Cr | 1.82 | 0.87 |
| Mn | 93.27 | 26.67 |
| Cu | 198.30 | 94.04 |
| Zn | 228.80 | 170.17 |
| Cd | 24.40 | 7.04 |
| Pb | NA | 29.62 |

**Supplemental Table S3: Estimated binding constants for the formation of ML_2_ complexes**

| Metal | logK_11_ | % error | logK_21_ | % error |
| --- | --- | --- | --- | --- |
| Cu^2+^ | 2.31 | 48.1 | 4.95 | 15.2 |
| Zn^2+^ | 5.71 | 195 | 4.58 | 170 |
| Mn^2+^ | 4.29 | 12.6 | 2.80 | 1.52 |
| Cd^2+^ | 5.15 | 72.9 | 5.35 | 59.3 |
| Pb^2+^ | 5.13 | 78.2 | 3.17 | 3.88 |

**Supplemental Table S4: Oligonucleotide primers used in RT-qPCR reactions**

| Gene | Fwd / Rev | Sequence | Notes | Annealing Temperature (℃) |
| --- | --- | --- | --- | --- |
| *MT1X* | Forward | 5’ -GCTTCTCCTTGCCTCGAAA -3’ | Metallothionein | 60 |
|  | Reverse | 5’ -TGACGTCCCTTTGCAGATG -3’ |  | 60 |
| *MT2A* | Forward | 5’- TCTTCAGCTCGCCATGGAT -3’ |  | 60 |
|  | Reverse | 5’- TTGTGGAAGTCGCGTTCTTTA -3’ |  | 60 |
| ***SOD1*** | Forward | 5’-GTACCACCATCCACTACAACCACATGTGTAACAGTTCCTGCAT -3’ | Superoxide dismutase | 60 |
|  | Reverse | 5’-ATGCAGGAACTGTTACACATGTGGTTGTAGTGGATGGTGGTAC-3’ |  | 60 |
| ***SOD2*** | Forward | 5’-GTAACAGTTCCTGCATGGGCGTCATGAACCGGAGGCCCATCCT-3’ | Superoxide dismutase | 60 |
|  | Reverse | 5’- AGGATGGGCCTCCGGTTCATGACGCCCATGCAGGAACTGTTAC-3’ |  | 60 |
| ***HO1*** | Forward | 5’- TTC AGA AGG GCC AGG TGA - 3’ | Heme Oxygenase | 60 |
|  | Reverse | 5’- TGT TGC GCT CAA TCT CCT C-3' |  | 60 |
| GAPDH | Forward | 5’- GCAGAGATGATGACCCTTTTGGCT -3’ | Reference Gene | 60 |
|  | Reverse | 5’- TGAAGCTCGGAGTCAACGGATTGGT -3’ |  | 60 |

**Supplemental Figure Legends**

**Figure S1** Ai-vi) Establishing toxic levels of each metal salt. Resazurin survival curves of Beas-2B cells incubated for 72 hrs with increasing concentrations of manganese, copper, zinc, chromium, cadmium and lead n=3. All error bars indicate SEM.

**Figure S2** A) Cell viability (measured by absolute resorufin fluorescence) of Beas-2B cells treated with increasing metals over 72 hrs, n=3. B) Beas-2B cells transfected with a Metal Response luciferase (MRE) and TK Renilla construct and incubated in mixed metal solution and each individual metal for 24 hrs. Luciferase values were normalised for Renilla expression, n=3. All error bars indicate SEM. * p<0.05, ** p<0.01, *** p<0.001, **** p<0.0001 relative to control.

**Figure S3** A) ICP-MS to determine metal levels in A549 cells treated for 24 hrs with mixed metal mix, n=3. B) Viability of A549 cells exposed to increasing metal concentrations C) ICP-MS to determine metal levels in H1417 cells treated for 24 hrs with mixed metal mix, n=3. All error bars indicate SEM. P ≤ 0.05, ** P ≤ 0.01, *** P ≤ 0.001, relative to control. D) Viability of H1417 cells exposed to increasing metal concentrations. E) Survival curves of H1417 cells exposed to increasing metal concentrations. All error bars indicate SEM.

**Figure S4** A) initial viability values derived from resazurin reduction assays for Beas-2B cells pre-incubated with metal mix for 24 hrs prior to treatment with doxorubicin for 72 hrs, n=3. B) Initial viability values derived from resazurin reduction assays for Beas-2B cells pre-incubated with individual metals for 24 hrs prior to treatment with doxorubicin for 72 hrs, n=3. C) Initial viability values derived from resazurin reduction assays for Beas-2B cells pre-incubated with a metal mix that was lacking one metal for 24 hrs prior to treatment with doxorubicin for 72 hrs, n=3. D) ICP-MS to determine the metal levels of Beas-2B cells treated for 24 hrs with each individual metal from the metal mix, as normalised by protein content, n=3. All error bars indicate SEM. Hashtags indicate T-test. * p<0.05, ** p<0.01, *** p<0.001, **** p<0.0001, relative to control conditions.

**Figure S5** (Ai-vi) ICP-MS to determine the metal levels of Beas-2B cells treated for 24 hrs with a metal mix that was lacking one of the metals as indicated, n=3. All error bars indicate SEM. * p<0.05, ** p<0.01, *** p<0.001, **** p<0.0001, black asterisks indicate statistics relative to control, grey relative to metal loaded conditions.

**Figure S6** A) Fold changes in *HO-1* mRNA expression were determined in Beas-2B cells exposed to 1X or 2X metals. *GAPDH* was used as reference gene and expression in the no metal control condition was set to 1. B-G ICP-MS to measure individual metal levels (i Manganese, ii copper, iii Zinc, iv Chromium, v Cadmium, vi Lead) in A549 cells exposed to 2X metals and NAC. C) MRE luciferase expression in A549 cells exposed to metals and NAC. Error bars represent SEM of n=3 * p<0.05, ** p<0.01, *** p<0.001, **** p<0.0001

**Figure S7** A) A549 cells were exposed to 6 or 12 metals and ROS levels examined using the Ros Glo kit (n=3). Bi) Fold change doxorubicin IC_50_ values of A549 cells exposed to either 6 or 12 metal mixes (n=3). Bii) Initial viabilities for A549 cells treated with the 6 or 12 metal mixes. C-D) qRT PCR mRNA expression of SOD1 (C) or SOD2 (D) in A549 cells exposed to all metals (2X), Copper, Manganese or Zinc. E ROS levels in A549 cells exposed to 2X metals or a metal mix lacking 1 metal, or exposed to Cu alone. Fi-ii) Doxorubicin IC_50_ survival values of A549 cells exposed to 2X metals, metal mix lacking 1 metal or Cu along and initial viabilities from this (ii). Error bars represent SEM of n=3 * p<0.05, ** p<0.01, *** p<0.001, **** p<0.0001, black asterisks indicate statistics relative to control, grey relative to metal loaded conditions.

**Figure S8** A) ROS levels in A549 cells exposed to medium or high levels of H_2_O_2_ and either 1:4 metals or the normal 2X metals as measured by a Ros-Glo kit. Bi) Viability of A549 cells exposed to medium or high levels of H_2_O_2_ and either 1:4 metals or the normal 2X metals. Bii) Doxorubicin IC_50_ values for A549 cells exposed to medium or high levels of H_2_O_2_ and either 1:4X metals or the normal 2X metals. C) ROS levels in A549 cells exposed to low or high levels of Menadione and either 1:4X, 1:2X, 1X metals or the normal 2X metals as measured by a Ros glo kit. Hashtags indicate T-test. Di) Viability of A549 cells exposed low or high levels of Menadione and either 1:4X, 1:2X, 1X metals or the normal 2X metals. Dii) Doxorubicin IC_50_ values for A549 cells exposed low or high levels of Menadione and increasing metals, derived from Di. Error bars represent SEM of n=3 * p<0.05, ** p<0.01, *** p<0.001, **** p<0.0001,

**Figure S9** A) Fold change IC_50_ values derived from resazurin reduction assays for Beas-2B cells preincubated for 24 hrs with metals prior to treatment with doxorubicin and four concentrations of EDTA for 72 hrs, n=3. B-C) Initial viability values derived from resazurin reduction assays for Beas-2B cells pre-incubated with metals for 24 hrs prior to treatment with doxorubicin and B) MiADMSA or C) EDTA for 72 hrs. D) Immunofluorescence images of doxorubicin uptake in Beas-2B cells exposed to metals and MiADMSA. E) quantification of doxorubicin intensity in cells of D.

F) *HO-*1 expression was determined in Beas-2B cells exposed to metals or MiADMSA after 24 or 72 hrs. All error bars indicate SEM. P ≤ 0.05, * p<0.05, ** p<0.01, *** p<0.001, **** p<0.0001, black asterisks indicate statistics relative to control, grey relative to metal loaded conditions.

**Figure S10** A) UV-Vis spectra of 1 mM MiADMSA in 0.1 M pH 7.4 HEPES buffer (black) titrated with CuSO_4_·5H_2_O in 0.05 equiv. increments up to 0.3 equiv. B) Changes in absorbance at 250 nm, 360 nm, 370 nm, 385 nm, 400 nm, and 410 nm for MiADMSA titrated with 0.05 equiv. increments of Cu^2+^, in the range of 0 – 0.3 equiv. C) Binding isotherm (top) and accompanying residuals (bottom) showing the changes in absorbance at selected wavelengths for the titration of MiADMSA with CuSO_4_·5H_2_O in the range of 0-0.3 equiv. Experimental absorbance values at the chosen wavelengths are represented as circles ( = 250 nm, = 360 nm, = 370 nm, = 385 nm, = 400 nm, = 410 nm) with the corresponding fitted BindFit model values shown as solid lines of the same colour. D) UV-Vis spectra of 1 mM MiADMSA in 0.1 M pH 7.4 HEPES buffer (black) titrated with ZnCl_2_ in 0.05 equiv. increments up to 1 equiv. E) Changes in absorbance at 300 nm, 320 nm, 340 nm, 350 nm, 360 nm, 380 nm and 400 nm for MiADMSA titrated with 0.05 equiv. increments of Zn^2+^, in the range of 0 – 1 equiv, and expansion of Figure S-7D in the wavelength range of 300 – 400 nm. F) Binding isotherm (top) and accompanying residuals (bottom) showing the changes in absorbance at selected wavelengths for the titration of MiADMSA with ZnCl_2_ in the range of 0-1 equiv. Experimental absorbance values at the chosen wavelengths are represented as circles ( = 300 nm, = 320 nm, = 340 nm, = 350 nm, = 360 nm, = 380 nm, = 400 nm) with the corresponding fitted BindFit model values shown as solid lines of the same colour.

**Figure S11** A) UV-Vis spectra of 1 mM MiADMSA in 0.1 M pH 7.4 HEPES buffer (black) titrated with MnCl_2_·4H_2_O in 0.05 equiv. increments up to 1 equiv. B) Changes in absorbance at 350 nm, 360 nm, 370 nm, 375 nm, and 380 nm for MiADMSA titrated with 0.05 equiv. increments of MnCl_2_·4H_2_O in the range of 0 – 1 equiv. C) Binding isotherm (top) and accompanying residuals (bottom) showing the changes in absorbance at selected wavelengths for the titration of MiADMSA with MnCl_2_·4H_2_O in the range of 0-1 equiv. Experimental values are represented as circles, with the corresponding fitted BindFit model shown as solid lines. Experimental absorbance values at the chosen wavelengths are represented as circles ( = 350 nm, = 360 nm, = 370 nm, = 375 nm, = 360 nm) with the corresponding fitted BindFit model values shown as solid lines of the same colour. D) UV-Vis spectra of 1 mM MiADMSA in 0.1 M pH 7.4 HEPES buffer (black) titrated with CdCl_2_·2.5H_2_O in 0.05 equiv. increments up to 1 equiv. E) Changes in absorbance at 250 nm, 280 nm, 300 nm, 320 nm, 340 nm, 360 nm and 380 nm for MiADMSA titrated with 0.05 equiv. increments of CdCl_2_·2.5H_2_O in the range of 0 – 1 equiv. F) Binding isotherm (top) and accompanying residuals (bottom) showing the changes in absorbance at selected wavelengths for the titration of MiADMSA with CdCl_2_·2.5H_2_O in the range of 0-1 equiv. Experimetal values are represented as circles, with the corresponding fitted BindFit model shown as solid lines. Experimental absorbance values at the chosen wavelengths are represented as circles ( = 250 nm, = 280 nm, = 300 nm, = 320 nm, = 340 nm, = 360 nm, = 380 nm) with the corresponding fitted BindFit model values shown as solid lines of the same colour.

**Figure S12** A) UV-Vis spectra of 1 mM MiADMSA in 0.1 M pH 7.4 HEPES buffer (black) titrated with Pb(OAc)_2_·3H_2_O in 0.05 equiv. increments up to 0.65 equiv. B) Changes in absorbance at 250 nm, 260 nm, 270 nm, 280 nm, 290 nm, 300 nm, 310 nm, 320 nm, and 340 nm for MiADMSA titrated with 0.05 equiv. increments of Pb(OAc)_2_·3H_2_O in the range of 0 – 0.65 equiv. C) Binding isotherm (top) and accompanying residuals (bottom) showing the changes in absorbance at selected wavelengths for the Pb^2+^-MiADMSA titration in the range of 0-1 equiv. Experimental absorbance values at the chosen wavelengths are represented as circles ( = 250 nm, = 260 nm, = 270 nm, = 280 nm, = 290 nm, = 300 nm, = 310 nm, = 320 nm, = 340 nm) with the corresponding fitted BindFit model values shown as solid lines of the same colour.

**Figure S13** Initial viability values derived from resazurin reduction assays for A) A549 cells or B) H1417 cells pre-incubated with metals for 24 hrs prior to treatment with  i, ii) doxorubicin or iii) etoposide and i, iii) MiADMSA  or ii) EDTA for 72 hrs. Initial viabilities are not treated with chemotherapeutics, n=3, all error bars indicate SEM.

**Supplemental Methods and Information**

**Data analysis/estimation of binding constants**

Estimations for the approximate values of binding constants were obtained by fitting multi-wavelength absorbance data from UV-Vis titrations to a 2:1 L-BFGS-B BindFit model. These binding constants, K_11_ and K_21_, describe the stepwise formation of the complex ML_2_, where M is a metal ion (Cu^2+^, Zn^2+^, Mn^2+^, Cd^2+^, or Pb^2+^) and L represents the ligand MiADMSA.

In the case of copper, binding to the HEPES buffer became apparent at higher metal concentrations (above 0.5 equiv.), therefore a limited range was used for modelling (0-0.3 equiv.) in which binding to MiADMSA dominates, as evidenced by the formation of the peak at 385 nm, characteristic of a Cu^2+^-thiolate LMCT transition.

For the titration of Pb(OAc)_2_ into MiADMSA, formation of a precipitate was visible from 0.7 equiv. Pb^2+^ onwards, likely due to the formation of insoluble aggregates, therefore only data from the range of 0-0.65 equiv. was used for estimation of binding constants through BindFit modelling. All absorbance values used in modelling calculations have been corrected for the effect of nitric acid on MiADMSA in the absence of metals

**NMR analysis**

For fluorine measurements, the background suppression pulse sequence scheme was:
